# Mesoporous silica nanoparticle-based nanomedicine: Preparation, functional modification, and theranostic applications

**DOI:** 10.1016/j.mtbio.2025.102223

**Published:** 2025-08-21

**Authors:** Yuying Liu, Man Zhao, Meihua Zhang, Bin Yang, Yun-Kun Qi, Qinrui Fu

**Affiliations:** a Key Laboratory of Maternal & Fetal Medicine of National Health Commission of China, Shandong Provincial Maternal and Child Health Care Hospital Affiliated to Qingdao University, Qingdao University, Jinan, 250014, China; b Institute for Translational Medicine, College of Medicine, Qingdao University, Qingdao, 266071, China; c School of Pharmacy, Qingdao University Medical College, Qingdao University, Qingdao, 266073, China; d Institute of Innovative Drugs, Qingdao University, Qingdao, 266021, China; e The Affiliated Hospital of Qingdao University, Qingdao, 266003, China

**Keywords:** Mesoporous silica nanoparticles, Synthesis strategies, Surface-functionalized modifications, Theranostic applications, Antibacterial applications, Vaccine applications

## Abstract

Mesoporous silica nanoparticles (MSNs) have emerged as cornerstone biomaterials for nanomedicine due to their highly tunable physicochemical properties. This review highlights recent breakthroughs in MSN-based platforms for disease theranostics. We systematically outline fundamental synthesis strategies and detail how surface functionalization-imparting properties such as targeting, stimuli-responsiveness, and biomimicry-transforms MSNs from passive carriers into intelligent theranostic agents. The review then summarizes the major contributions of these engineered platforms across key diagnostic (*e.g.*, advanced imaging) and therapeutic (*e.g.*, targeted drug delivery, dynamic therapies) applications, underscoring their potential in tackling complex diseases. Finally, we address the critical challenges hindering clinical translation, including manufacturing scalability and long-term biocompatibility. We conclude by outlining future directions, such as integration with emerging modalities like immunotherapy, aimed at accelerating the bench-to-bedside transition of MSN-based nanomedicines.

## Highlights

• A comprehensive overview of MSN synthesis and surface engineering.
• The Broad-spectrum biomedical applications of MSNs-based nanoplatforms.
• Translational insight and future prospects of MSNs for clinical application.

## Graphical abstract

**Mesoporous Silica Nanoparticle-Based Nanomedicine: Preparation, Functional Modification, and Theranostic Applications** comprehensively summarizes the most recent advancements in mesoporous silica nanoparticles (MSNs) for disease theranostics, systematically discussing innovative synthesis approaches, advanced surface functionalization strategies, and their extensive biomedical applications. Furthermore, it critically examines current challenges and provides insightful perspectives on future directions to facilitate clinical translation.

**Figure:**
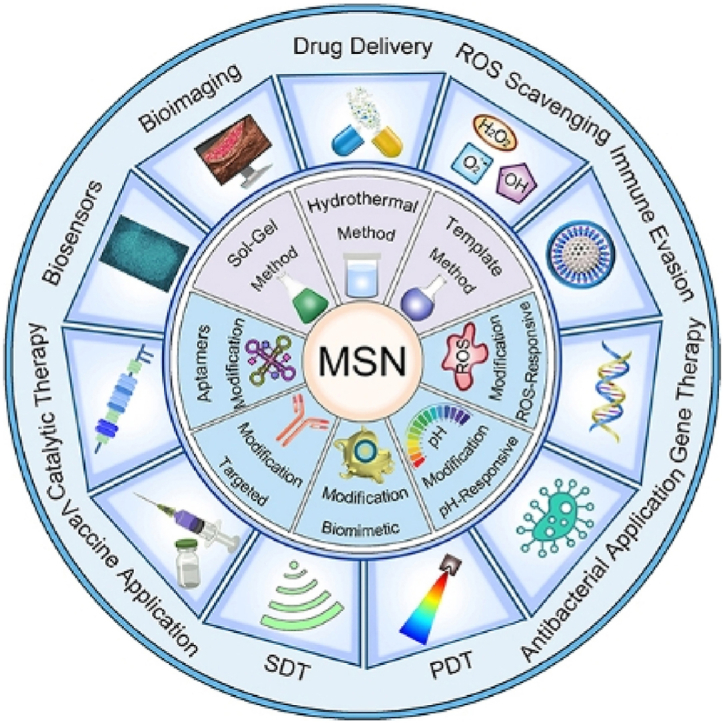

## 1 Introduction

With the rapid progress of nanotechnology, nanomaterials featuring excellent physicochemical properties find extensive applications in biomedicine. Considerable efforts have been dedicated to the design of multifunctional nanoformulations with customized capabilities for precise cargo delivery, advanced diagnostics, and targeted therapeutics [[1], [2], [3], [4], [5], [6], [7], [8], [9], [10], [11], [12]]. Nanoformulations present superior pharmaceutical advantages when compared to conventional macroformulations. These benefits include enhanced bioavailability, reduced systemic toxicity - a critical advantage considering the significant hematological and histopathological side effects associated with many conventional drugs like diclofenac sodium [13] - and improved *in vivo* target selectivity [14]. Nanotherapeutics are typically classified into two main types: organic and inorganic nanoformulations [15]. The clinical translation of organic nanocarriers has been convincingly evidenced by two significant achievements: i) The landmark approval of Doxil® in 1995, which confirmed liposomes as viable clinical delivery systems [16]. ii) The emergency authorization of lipid nanoparticles-based COVID-19 mRNA vaccines (BNT162b2 and mRNA-1273), marking a paradigm shift in vaccinology [17]. Beyond these synthetic organic systems, researchers are also exploring novel biological carriers, such as drug-loaded living organisms, for the targeted treatment of challenging diseases like gastrointestinal cancer [18]. Although the inherent biocompatibility of some inorganic nanoparticles (NPs) can pose greater challenges compared to their organic counterparts, it is crucial to discuss this with nuance. The toxicity concerns associated with MSNs are complex and can be significantly mitigated through strategic functionalization, a point corroborated by recent research. For instance, studies have shown that appropriately modified MSN-based nanoplatforms can serve as highly effective and biocompatible theranostic agents for treating complex inflammatory diseases [19]. The ease and versatility of functionalization have thus become a cornerstone of MSN-based nanomedicine, enabling precise control over their biological interactions and therapeutic outcomes [20]. Consequently, these engineered inorganic platforms often demonstrate greater superiority in stability and drug delivery efficiency. Inorganic NPs have effectively facilitated the development and application of various innovative nanotherapeutic methods owing to their unique optical, ultrasonic, magnetic, and catalytic properties. These methods include photothermal therapy (PTT), photodynamic therapy (PDT), sonodynamic therapy (SDT), chemodynamic therapy (CDT), and nanozyme-based catalytic therapy, which are crucial therapeutic strategies [[21], [22], [23], [24], [25], [26], [27], [28], [29], [30]]. Encouragingly, inorganic NPs have gradually entered the field of clinical medicine, with approximately 25 inorganic nanomedicines currently approved for clinical use [31]. A prominent example is the development of Cornell dots (C′ dots), ultrasmall silica-based fluorescent nanoparticles, which became the first of their kind to be approved by the FDA for clinical trials in cancer imaging [32]. The successful clinical translation of such silica-based platforms provides a strong precedent and valuable insights for the development of more complex mesoporous silica-based systems, which are the focus of this review. This progress is built upon extensive research into a wide array of inorganic nanomaterials. For example, significant efforts are dedicated to the green synthesis of silver nanoparticles for their antibacterial and cytotoxic potential [33], and the development of sophisticated magnetic nanocomposites supported on materials like activated carbon for applications in antimicrobial therapy and environmental remediation [34,35].

Among a variety of inorganic nanocarriers, mesoporous silica nanoparticles (MSNs) have attracted extensive global research interest owing to a unique combination of structural and morphological characteristics that enable them exceptionally suitable as nanomedicine platforms [[36], [37], [38]]. The widespread application of MSNs in the biomedical field is rooted in the following key attributes: 1) High Specific Surface Area and Large Pore Volume: The intrinsic mesoporous network provides MSNs with an exceptionally large surface area (often >700 m^2^/g) and pore volume [39]. This structural feature is paramount for achieving high loading capacity for a wide range of therapeutic agents, including small-molecule drugs, peptides, and genes, thereby enhancing therapeutic efficacy. 2) Tunable Pore Size: The diameter of the mesopores (typically 2–50 nm) can be precisely engineered by selecting different templates during synthesis [40]. This tunability allows for the tailored encapsulation of guest molecules based on their size and prevents premature leakage, enabling sophisticated control over drug release kinetics. 3) Controllable Particle Size and Morphology: The overall particle size of MSNs (typically 50–200 nm) can be meticulously controlled, which is crucial for modulating their *in vivo* behavior, such as blood circulation time, biodistribution, and cellular uptake [41]. This control is vital for leveraging passive targeting mechanisms like the enhanced permeability and retention (EPR) effect in tumors. Furthermore, their morphology (*e.g.*, spherical, rod-like) can also be adjusted to influence cellular interactions and therapeutic outcomes [42]. 4) Versatile Surface Chemistry: The surface of MSNs is rich in silanol groups (-*Si*-OH), which serve as readily available anchor points for functional modification [43]. This chemical versatility facilitates the covalent grafting of targeting ligands (*e.g.*, antibodies, peptides), stimuli-responsive “gatekeepers," and stealth polymers (*e.g.*, PEG), transforming MSNs into intelligent, targeted theranostic agents [44]. Collectively, these distinct structural and physicochemical properties endow MSNs with unparalleled design flexibility, enabling them a cornerstone material in the development of next-generation nanomedicine.

The evolution from simple nanoparticle synthesis to sophisticated *in vivo* applications represents a valuable paradigm shift in the field. Within the realm of inorganic nanoparticles, a key contemporary focus is on microstructured nanoparticles. Recent advances, for example, have highlighted the power of integrating these inorganic cores with smart polymers to create hybrid systems for stimuli-responsive drug delivery, which have shown significant promise in overcoming formidable biological barriers like the blood-brain barrier [45]. Beyond functional sophistication, the field is also moving towards greater sustainability and translatability. This includes developing protocols for the renewable synthesis of MSNs, standardizing their characterization, and optimizing loading protocols for a diverse range of therapeutics, from small molecules to proteins and nucleic acids like siRNA and mRNA [46]. These trends underscore a maturation of the field, moving towards more robust, reproducible, and clinically viable nanomedicines. It is in this advanced context that MSNs, as a prototypical microstructured nanoparticle, truly shine.

The precisely controllable fabrication and straightforward functionalization of MSNs endow these intricate nanosystems with unique physicochemical characteristics. This confers upon them a high degree of adaptability to specific circumstances. For instance, owing to their multifunctional nature and outstanding biocompatibility, MSNs can serve as robust nanoplatforms for biomedical applications within complex biological milieus [[47], [48], [49]]. Consequently, the current research trajectory in the realm of mesoporous materials is gradually transitioning from a primary emphasis on microstructure-controlled synthesis methodologies to a more in-depth exploration of their practical *in vivo* applications in the medical domain [[50], [51], [52], [53], [54]]. The large specific surface area and pore diameter of MSNs facilitate the efficient delivery of drugs or other therapeutic molecules to the surrounding biological environments, thereby substantially enhancing the treatment efficacy. At present, MSNs have been extensively employed across a wide range of therapeutic areas, encompassing controlled drug release, gene delivery, diagnostic imaging, and tissue engineering applications [[55], [56], [57]]. These versatile nanoplatforms display an extraordinary diversity in terms of size, morphology, architecture, composition, and functionality, enabling a customized design that can be tailored to meet specific biomedical requirements. Such structural flexibility significantly broadens the scope of therapeutic and diagnostic applications in the field of nanomedicine.

**Fig. 1:**
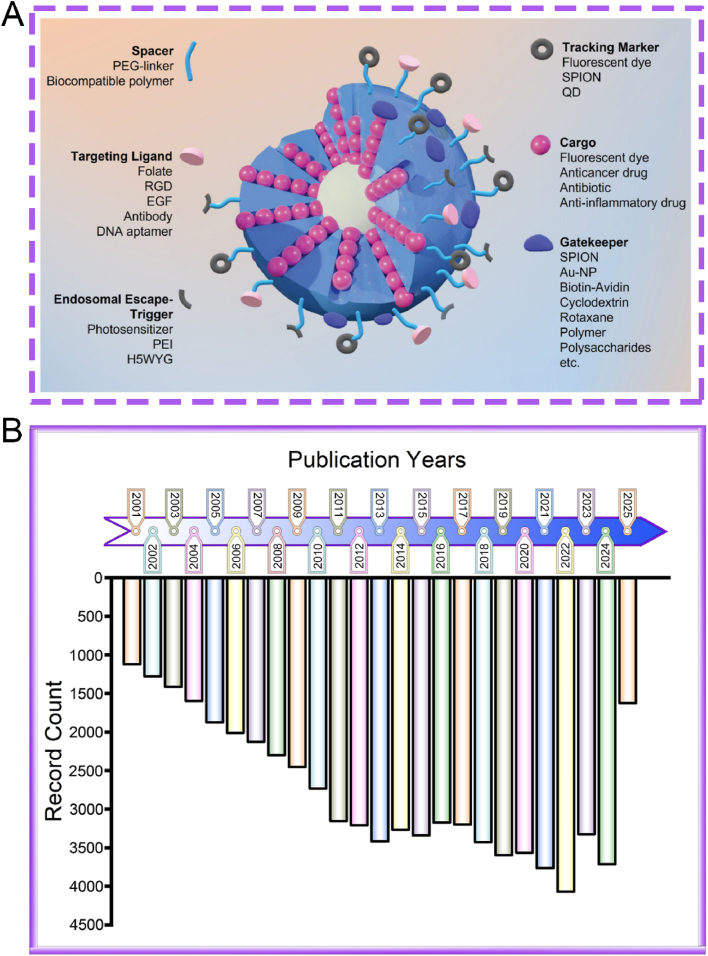
(A) Schematic mechanism diagram of functional modification of MSN. (B) Schematic illustration showing the published literature on the application of silicon dioxide in biomedical fields, derived from the analysis by Web of Science. (A) Reproduced with permission [61]. Copyright 2025, Whily. (For interpretation of the references to color in this figure legend, the reader is referred to the Web version of this article.)

**Scheme 1:**
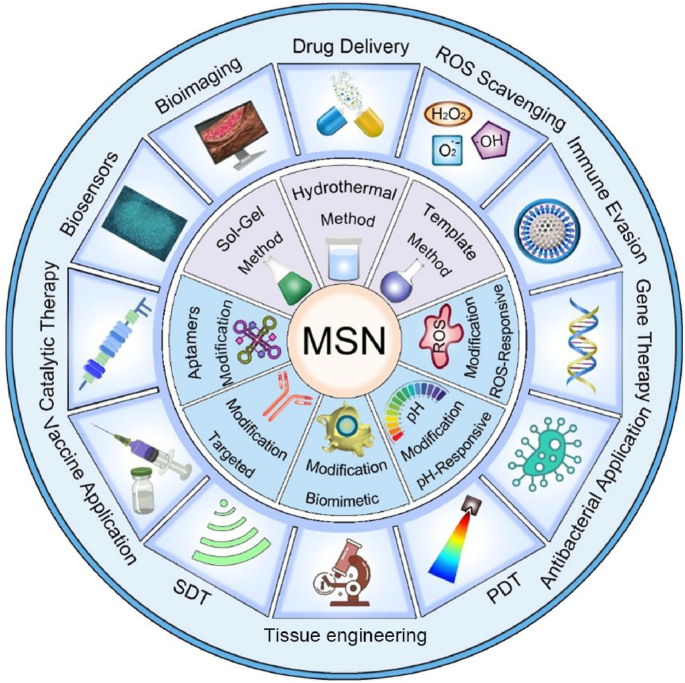
Schematic illustration of the synthesis, functional modification, and theranostic applications of nano-enabled mesoporous silica materials.

A number of review articles have recently reported on the advances of mesoporous silica-based nanoplatforms in nanodynamic therapy and photo-activated tumor treatment [58,59]. Additionally, some publications have explored their applications in tumor theranostics, particularly from the perspective of biodegradability [60]. However, to date, there remains a lack of a comprehensive review that systematically summarizes the preparation strategies, surface functionalization methods, and biomedical theranostic applications of mesoporous silica-based nanoplatforms. Therefore, this review offers a more comprehensive integration of recent breakthroughs from preparation strategies, functionalized modification and theranostic applications. In this review, we systematically summarize the fundamental synthesis methods of MSNs, encompassing the sol-gel method, hydrothermal method, and template method. Subsequently, we elaborate on several functionalized modification strategies for MSNs, such as targeted modification, biomimetic modification, stimulus-responsive modification, and aptamer-based surface modification. Additionally, we present a concentrated overview of the latest advancements in MSN-guided biomedical applications, placing particular emphasis on their diagnostic and therapeutic applications across a diverse range of diseases. Finally, we rigorously assess the current clinical translation status of MSNs and tackle the primary obstacles impeding their full-fledged biomedical implementation. This review provides a systematic overview of strategies to optimize MSN-based nanoplatforms, with the goal of enhancing their clinical utility and translational potential. While we adopt a foundational framework that logically progresses from preparation to application, our primary aim is to provide a distinctive, theranostic-centric perspective. To demonstrate these theranostic applications aesthetically, Fig. 1A depicts the functionalized mechanisms of the MSN platform schematically. The functionalization predominantly comprises: therapeutic cargoes, responsive gates, targeting ligands, tracking markers, and endosomal escape triggers [61]. Although the biomedical applications of silicon dioxides are vast in the recent two decades (Fig. 1B), this review will place particular emphasis on MSNs roles in the theranostics of cancer and inflammatory diseases. This focus is deliberate, as recent scholarship highlights the value of contextualizing nanotechnologies within specific disease landscapes, where MSNs have demonstrated remarkable progress as versatile platforms for both enhancing cancer theranostics [62] and for treating inflammatory disorders [19]. By narrowing our scope to these highly active research fronts, we seek to systematically elucidate the connections between fundamental synthesis methods and their influence on surface modification potential, and further, how these refined modifications empower the advanced theranostic applications at the forefront of modern nanomedicine. This purpose-driven narrative, visually summarized in Scheme 1, is designed to serve as an insightful roadmap for the rational design of next-generation MSN-based theranostics.

## 2 Preparation strategies

**Fig. 2:**
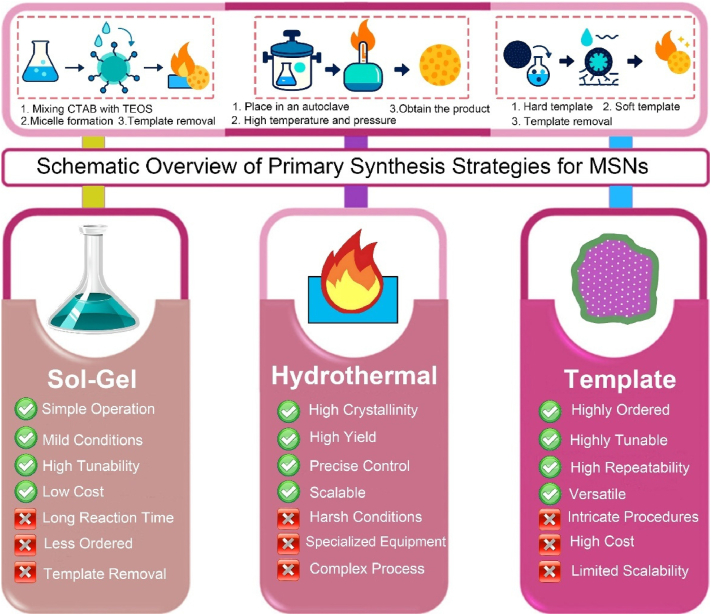
Comparative schematic of the primary strategies for the fabrication of Mesoporous Silica Nanoparticles (MSNs). The figure illustrates three representative fabrication methods-Sol-Gel, Hydrothermal, and Template-each with its characteristic workflow, advantages, and limitations. The Sol-Gel method features mild reaction conditions and high tunability but suffers from long reaction times and lower structural order. The Hydrothermal method enables high crystallinity and scalability under harsh conditions requiring specialized equipment. The Template method offers precise structural control and versatility but involves intricate procedures and limited scalability. This comparative schematic provides a concise visual summary to guide the selection of appropriate synthesis strategies for specific biomedical applications.

MSNs are regarded as a subset of inorganic nanoparticles. They are characterized by their ordered mesoporous structure, which mainly consists of a silicon dioxide (SiO_2_) framework. MSNs have attracted significant attention in the domains of drug delivery, biosensing, and disease theranostics [[63], [64], [65]]. Leveraging these properties, MSNs can encapsulate and deliver a wide variety of therapeutic agents, including nucleic acids, drugs, and CRISPR-based gene-editing tools [66]. To enhance drug delivery and theranostic applications, MSNs are expected to possess adjustable shape, particle size, surface charge, and pore structure through various preparation methods [[67], [68], [69], [70], [71]]. This section presents a comprehensive overview of diverse synthesis strategies for mesoporous silica nanoparticles (MSNs), encompassing the sol-gel method, hydrothermal method, and template method. A visual summary of these strategies, highlighting their core mechanisms and respective pros and cons, is provided in Fig. 2. The advantages and disadvantages of these three methods have been systematically summarized in Table 1.

**Table 1:** Comparative analysis of primary strategies for the fabrication of mesoporous silica nanoparticles (MSNs).

Strategy	Sol-Gel Method	Hydrothermal Method	Template Method
Key Advantages	The operation is simple, the conditions are mild, and the tunability is high.	Exhibiting high crystallinity, remarkable stability, and a high yield.	Highly ordered mesoporous structure, along with tunable pore size and morphology.
Key Disadvantages	A relatively long reaction time, along with the removal of the template agent.	Equipment operating under high temperature and high pressure is subject to relatively stringent reaction conditions.	The removal of the template agent entails intricate procedures.
Typical Particle Size (nm)	50–300	100–500	50–1000
Typical Pore Size (nm)	2–15	2–10	2-50∼
Yield/Scalability	Moderate yield; Good lab-scale scalability	High yield (>90 %); Good potential for industrial scale-up	Variable yield; Generally lower scalability, especially for hard templates
Refs.	[[72], [73], [74]]	[[75], [76], [77], [78]]	[[79], [80], [81]]

### 2.1 Sol-gel method

The sol-gel technique, a quintessential example of soft-templating in soft chemistry, is widely recognized for the synthesis of MSNs. In this inorganic polymerization process, soluble metal alkoxide precursors-most commonly tetraethyl orthosilicate (TEOS)-are converted into solid metal oxides with three-dimensional networks through a series of hydrolysis and condensation reactions, typically conducted under mild conditions (room temperature or slightly elevated) [[82], [83], [84], [85], [86], [87]]. The process initiates with the self-assembly of surfactant molecules, such as the cationic surfactant cetyltrimethylammonium bromide (CTAB), forming ordered micellar structures (*e.g.*, cylindrical rods) in aqueous solution. Upon introduction of the silica precursor (TEOS), hydrolysis and condensation yield silica species that co-assemble around the surfactant micelles via electrostatic interactions, resulting in a rigid inorganic silica framework that mimics the ordered structure of the template. Additional reagents, such as 3-aminopropyltriethoxysilane (APTES), can be employed to introduce functional groups [[88], [89], [90]]. The final step involves removal of the surfactant template-typically through high-temperature calcination or solvent extraction-producing highly ordered mesoporous silica networks with particle sizes generally ranging from 60 to 100 nm. The aerosol-assisted sol-gel method is an advanced technique in which the precursor solution is atomized into fine droplets and passed through a heated furnace. Each droplet serves as a microreactor, enabling rapid hydrolysis, condensation, and self-assembly. This approach streamlines the synthesis process, allowing for continuous and efficient production of highly spherical and uniform MSNs, often integrating synthesis and template removal into a single step. Whether through the conventional batch process or the continuous aerosol-assisted route, this versatile approach enables precise control over pore size, morphology, and surface properties, establishing it as a foundational and widely adopted strategy for the preparation of MSNs.

**Fig. 3:**
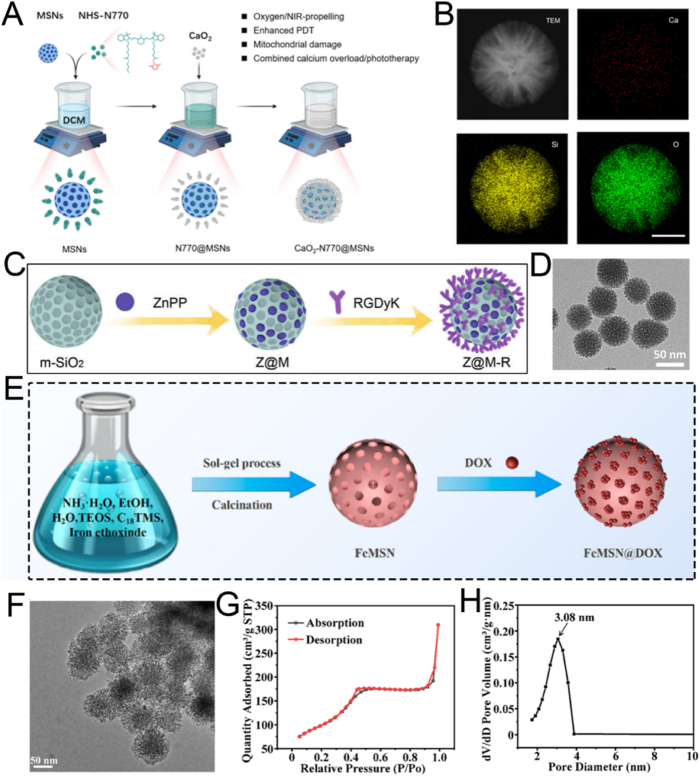
(A) Schematic illustration of the fabrication procedure of CaO_2_-N770@MSNs. (B) Transmission electron microscope (TEM) and Energy dispersive spectrometer (EDS) characterization of CaO_2_-N770@MSNs. Scale bar: 200 μm. (C) Schematic diagram of ZnPP@MSN-RGDyK(Z@M-R) fabrication. (D) Representative TEM image of Z@M-R. (E) Schematic illustration of the FeMSN@DOX preparation. (F) Representative TEM image of FeMSN@DOX. (G) N_2_ adsorption-desorption isotherm of FeMSN@DOX. (H) Appropriate pore size distribution of FeMSN@DOX. (A, B) Reproduced with permission [72]. Copyright 2023, Elsevier. (C, D) Reproduced with permission [73]. Copyright 2022, Wiley Online Library. (E–H) Reproduced with permission [74]. Copyright 2022, Elsevier.

For instance, Xiao et al. employed CTAB as the mesoporous structure-directing agent, TEOS as the silica precursor, and mitochondrial N770 to fabricate calcium peroxide (CaO_2_)-conjugated mesoporous silica nanoparticles (CaO_2_-N770@MSNs) *via* the sol-gel method for photo-immunotherapy against colorectal cancer (Fig. 3A) [72]. Briefly, CTAB, NH_3_·H_2_O, ethyl ether, and ethanol were mixed and reacted thoroughly for 0.5 h. Subsequently, TEOS was added and stirred vigorously for an additional 4 h to obtain MSNs. Then, N770 was added to amino group-functionalized MSNs based on electrostatic adsorption to fabricate the final N770@MSNs material. The synthesized N770@MSNs exhibited a branched mesoporous structure with a diameter of 400 nm and a pore size of 4.58 nm, enabling efficient loading of CaO_2_ (Fig. 3B). The incorporation of CaO_2_ endowed N770@MSNs with calcium overload characteristics, which could be further enhanced by phototherapy performance, thus facilitating synergistic calcium overload and phototherapy. Although this therapeutic platform (CaO_2_-N770@MSNs combined with NIR irradiation and αPD-L1) has demonstrated both biosafety and efficacy, its preparation involves multiple steps, which may pose challenges for clinical translation. Therefore, further optimization and simplification of the preparation process are necessary to better meet the requirements of practical clinical applications.

Some patients with non-small cell lung cancer spinal metastasis are unresponsive to immune checkpoint inhibitors (ICIs) targeting programmed death 1 (PD 1)/programmed death ligand 1 (PD-L1). To tackle this problem, Jiang and colleagues designed a drug delivery nanocarrier, ZnPP@MSN-RGDyK, targeting NSCLC-SM with PD-L1. This nanocarrier consists of zinc protoporphyrin (ZnPP), MSN, and RGDyK and is used for non-small cell lung cancer spinal metastasis PDT and immunotherapy (Fig. 3C) [73]. Among these components, the MSN nanoparticles were prepared *via* the sol−gel method. Briefly, cetyltrimethylammonium chloride (CTAC), CH_3_COONa, and double-distilled water were vigorously stirred at 60 °C for 1 h. Then, TEOS was added to the above solution and continuously stirred for 16 h. Subsequently, APTES was added and stirred for an additional 4 h to synthesize MSN. These synthesized MSN nanoparticles exhibited a characteristic spherical mesoporous structure with a diameter of 50 nm and excellent monodispersity of mesoporous nanospheres (Fig. 3D). To boost the synergistic effect of PDT and ICIs by releasing tumor antigens to activate T cells, the integrin β3 inhibitor RGDyK (which promotes PD-L1 ubiquitination) and ZnPP were encapsulated in MSNs. Overall, the ZnPP@MSN-RGDyK ultimately synthesized *via* the sol-gel method demonstrated excellent PDT efficiency and remarkable immunosuppressive effects.

As well as conventional MSN, mesoporous nanozymes have attracted significant attention for their applications in drug delivery and disease theranostics. These nanozymes not only exhibit enzyme-mimicking catalytic functions but also integrate with other therapeutic modalities to enable efficient disease theranostics. For example, He et al. utilized TEOS as a silica precursor and octadecyl trimethoxysilane (C18TMS) as a mesoporous structure aligner to fabricate iron-doped mesoporous silica nanoparticles (FeMSNs) *via* a sol-gel method for synergistic CDT and chemotherapy (Fig. 3E) [74]. Briefly, iron ethoxide, C18TMS, and TEOS were homogeneously mixed and allowed to react at 30 °C for 6 h. Subsequently, the resultant product was calcined at 550 °C for 6 h to obtain the final FeMSNs material. The fabricated FeMSNs exhibited a spherical mesoporous morphology with a diameter of 80 nm and a pore size of 3.08 nm, which facilitated the efficient incorporation of the chemotherapeutic drug doxorubicin (Dox) (Fig. 3F–H). The electrostatic interaction between FeMSNs and Dox enabled the assembly of Dox-loaded FeMSNs (FeMSN@Dox). The incorporation of iron endowed FeMSNs with peroxidase-like properties, which could synergize with Dox chemotherapy.

### 2.2 Hydrothermal method

**Fig. 4:**
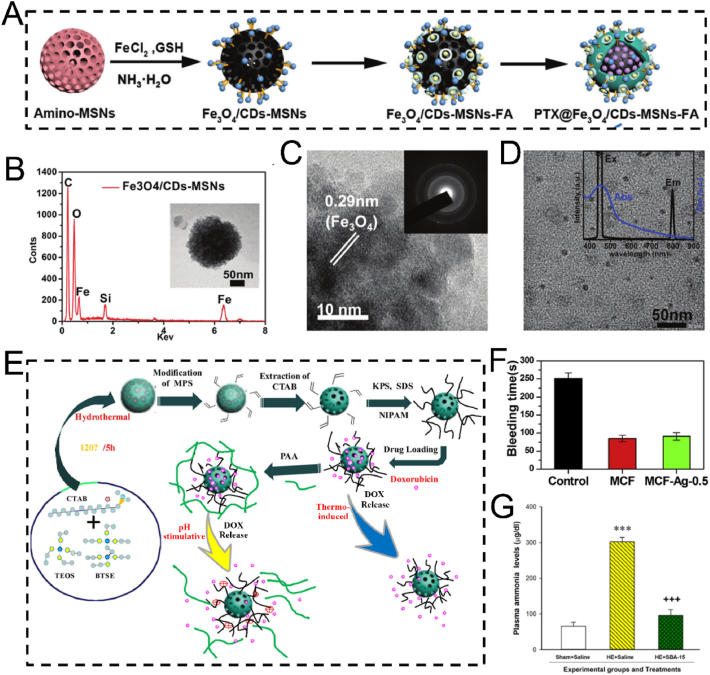
(A) Schematic illustration of the PTX@Fe_3_O_4_/CDs-MSNs-FA synthesis. (B) EDS analysis and a TEM image of Fe_3_O_4_/CDs-MSNs. (C) A high-power TEM picture and X-ray diffraction image of a local area containing Fe_3_O_4_/CDs-MSNs, showing the presence of Fe_3_O_4_. (D) TEM images and fluorescence spectra of CDs derived from Fe_3_O_4_/CDs-MSNs after destroying the silica matrix and Fe_3_O_4_ with sodium hydroxide and hydrochloric acid. (E) Schematic illustration of the preparation and drug release control mechanism of a thermo/pH-responsive drug delivery platform comprising hollow hybrid mesoporous silica nanoparticles. (F) Statistical evaluation of bleeding time for rabbit live body rupture injuries in different groups. (G) Comparison of ammonia levels in plasma among all experimental groups. (A–D) Reproduced with permission [75]. Copyright 2021, The Royal Society of Chemistry. (E) Reproduced with permission [76]. Copyright 2020, Elsevier. (F) Reproduced with permission [77]. Copyright 2020, Elsevier. (G) Reproduced with permission [78]. Copyright 2020, Mashhad University of Medical Sciences.

The hydrothermal technique is an advanced synthetic method for producing MSNs, in which raw materials are dissolved in an aqueous solution and subjected to high-temperature, high-pressure conditions within a Teflon-lined stainless-steel autoclave [[91], [92], [93]]. Typically, the reaction mixture-comprising a silicon precursor and a surfactant template-is heated to 100–180 °C for an extended period. A significant enhancement to this process is the microwave-assisted hydrothermal method, which utilizes microwave irradiation instead of conventional oven heating. This approach dramatically reduces reaction times, often from hours to mere minutes, by providing rapid, uniform, and direct energy to the reaction mixture, thereby improving energy efficiency and synthesis speed. Compared to the conventional sol-gel method, the hydrothermal process accelerates the hydrolysis and condensation of the silica framework, resulting in a higher degree of cross-linking and the formation of thicker, more robust pore walls [[94], [95], [96], [97], [98]]. The elevated temperature and autogenous pressure also facilitate the dissolution and re-deposition of silica species (akin to Ostwald ripening), effectively healing structural defects and yielding MSNs with exceptional crystallinity, improved long-range porous order, and well-defined morphologies. The hydrothermal method is highly versatile, enabling precise control over MSN morphology and porosity by adjusting parameters such as pH, temperature, reaction time, and surfactant type. 1) pH: High pH speeds up silica formation and produces uniform, spherical particles; lower pH creates more complex shapes like rods or sheets. 2) Temperature: Higher temperatures and longer durations yield larger, more stable particles, but excessive heating can cause aggregation. 3) Surfactant: The type and amount of surfactant determine pore size-ationic surfactants produce smaller pores, while non-ionic ones create larger pores suitable for loading bigger molecules. By tuning these factors, researchers are available to customize MSN structures for specific drug delivery and theranostic uses. For instance, larger pore sizes (>10 nm) achieved through specific surfactants are crucial for accommodating bulky therapeutic cargo like proteins and nucleic acids, while precise control over morphology to create hollow structures can dramatically increase the drug loading capacity. This enables hydrothermal synthesis a valuable tool in nanomedicine. Chen et al. synthesized the tumor-targeted and tumor-responsive MSNs-based nanocomposite, Fe_3_O_4_/CDs-MSNs-FA, *via* a straightforward hydrothermal approach for tumor-selective multimodal theranostics (Fig. 4A) [75]. The fabricated Fe_3_O_4_/CDs-MSNs-FA functioned as a tumor-targeting agent, facilitating imaging-mediated synergistic chemo-catalytic-photothermal therapy. Specifically, Fe_3_O_4_ and carbon dots were introduced *in situ* through a hydrothermal method by coordinating with Fe^2+^ and glutathione (GSH), thereby endowing MSNs with multimodal theranostic capabilities (Fig. 4B–D). Fe_3_O_4_ acted as both a photothermal agent and a catalyst, while carbon dots were instrumental in fluorescence imaging. After loading paclitaxel (PTX), polyester and folate-conjugated cyclodextrin were utilized as an esterase-sensitive gatekeeper to regulate the release of PTX from the pores of MSNs and as a tumor-specific agent for precise therapy, respectively. As expected, the nanoplatform effectively accumulated in tumor cells, followed by synergistic chemo-catalytic-photothermal therapy, leading to an apoptosis rate in tumor cells that was five times higher than that in healthy cells. *In vivo* experiments evidenced significant tumor suppression, and the survival rate of mice treated with the nanocomposite increased to over 80 % after five weeks of treatment. Preliminary biosafety of this nanoplatform was verified by monitoring the body weight, major organ weight, and histological staining of mice. However, systematic evaluations of its long-term metabolic behavior *in vivo* (beyond five weeks), the potential toxicity of degradation products, and immunogenicity remain to be conducted. In conclusion, this hydrothermally fabricated MSN-based nanocomposite presents a promising theranostic strategy for tumor therapy, though further studies are required to comprehensively assess its long-term biosafety and clinical translation potential.

Developing drug delivery nanoplatforms based on hollow mesoporous silica nanoparticles still poses considerable difficulties. To tackle this issue, He et al. successfully fabricated polyacrylic acid encapsulated hollow hybrid mesoporous silica to specifically deliver Dox for the anticancer therapy through a one-step hydrothermal method (Fig. 4E) [76]. Specifically, the pore-forming template CTAB was dissolved in a mixed solution of ammonia, ethanol, and water, and stirred at 35 °C for 60 min. Subsequently, two silica sources, TEOS and 1,2-Bis(triethoxysilyl)ethane (BTSE), were rapidly added, followed by continuous stirring at 1100 rpm at 35 °C for 24 h. The resulting product was collected by centrifugation and washed three times with ethanol. The obtained hybrid MSNs were then dispersed in water and transferred to a stainless-steel autoclave lined with polytetrafluoroethylene, where the hydrothermal reaction was carried out at 120 °C for 5 h. Owing to the hydrothermal treatment, the MSNs underwent a transformation from a solid to a hollow structure. The innovative hollow hybrid MSN prepared by hydrothermal method demonstrated exceptional biocompatibility and superior drug-loading capacity, highlighting its significant potential for application in cancer therapy. In addition to its exceptional performance in cancer therapy, the hydrothermally optimized MSN-based nanoplatform also demonstrated excellent efficacy in biomedical applications such as antibacterial hemostasis and the alleviation of hyperammonemia (Fig. 4F and G) [77,78]. These results conclusively emphasized its broad potential as a versatile nanotheranostic platform.

### 2.3 Template method

The template method for synthesizing MSNs commonly employs surfactants (such as CTAB, CTAC, and Pluronic F127) as structure-directing agents. These surfactants self-assemble to form mesoporous frameworks, into which a silicon source is introduced and guided to organize into ordered mesoporous structures [[99], [100], [101], [102]]. After synthesis, the template is removed to yield pure MSNs [103,104]. In recent years, growing attention has been given to novel template agents and green synthesis approaches, which aim to improve sustainability and reduce environmental impact. For example, natural resources such as diatom-derived silica have been explored as bio-templates, offering renewable and environmentally friendly alternatives to conventional surfactants [105,106]. These emerging strategies broaden the scope of MSN synthesis and highlight the potential of green chemistry in this field.

**Fig. 5:**
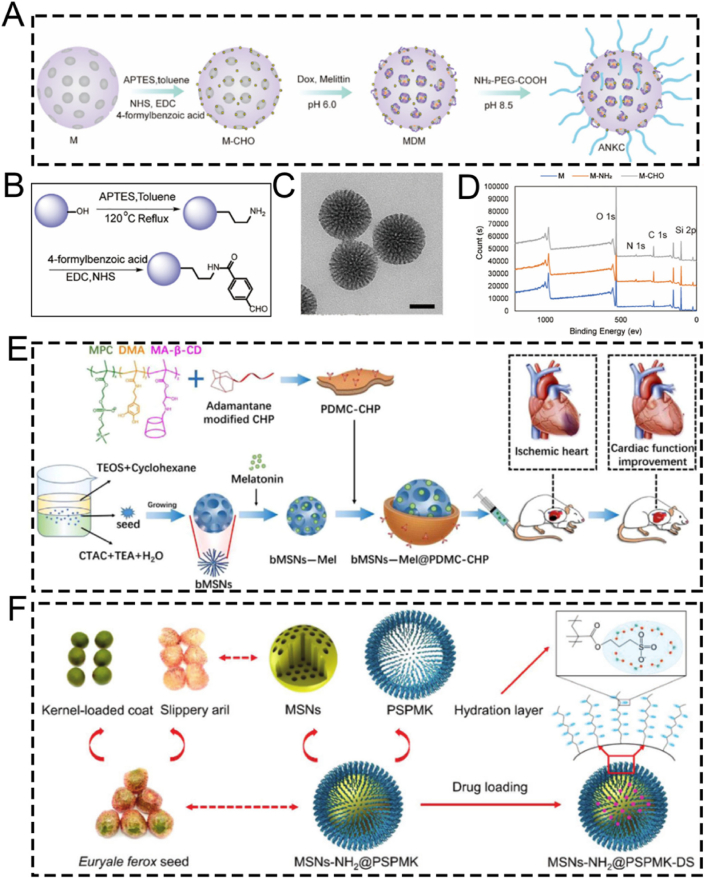
(A) Schematic diagram of ANKC construction. (B) Schematic representation of the M-CHO synthesis. (C) TEM image of M-CHO, scale bar: 50 nm. (D) X-ray photoelectron spectra (XPS) of M, M − NH_2_ and M-CHO. (E) Schematic diagram revealing the fabrication process of bMSNs-Mel@PDMC-CHP and the corresponding therapeutic effect of functionalized nanoparticles *via* intraventricular injection in mice with acute myocardial infarction. (F) Schematic diagram of the fabrication of ultra-lubricated drug-carrying nanoparticles MSNs-NH_2_@PSPMK-DS. (A–D) Reproduced with permission [79]. Copyright 2022, Elsevier. (E) Reproduced with permission [80]. Copyright 2024, Wiley. (F) Reproduced with permission [81]. Copyright 2018, Wiley.

For instance, Mei et al. designed a pH-labile benzaldehyde-functionalized MSN as a scaffold material for artificial natural killer cells (ANKC). They employed CTAC and triethanolamine (TEA) as templates. The antitumor drugs Dox and melittin were loaded into the pores of the MSNs to overcome tumor drug resistance (Fig. 5A) [79]. Subsequently, the MSNs were successively functionalized with amino and aldehyde groups to facilitate drug loading (Fig. 5B). Transmission electron microscopy (TEM) characterization confirmed that the functionalized MSNs had a mesoporous structure (Fig. 5C). X-ray photoelectron spectroscopy (XPS) elemental analysis verified the successful modification of MSNs with APTES, enabling the attachment of 4-carboxybenzaldehyde to the MSNs *via* amide bonds (Fig. 5D). Subsequently, Dox and melittin were loaded onto the 4-carboxybenzaldehyde-functionalized MSNs through Schiff base formation under weakly alkaline conditions. The pH-sensitive ANKC released Dox and melittin in the weakly acidic tumor microenvironment. Similar to perforin, melittin forms pores on the plasma membrane and endosomes, ensuring the intracellular transport of Dox. Meanwhile, Dox, similar to granzymes, initiates tumor cell apoptosis. In conclusion, MSNs fabricated using the template method and loaded with melittin and Dox present a promising strategy for treating drug-resistant tumors.

Apart from their applications in certain tumor diseases, MSNs fabricated through the template method are also employed in cardiovascular diseases, such as myocardial infarction. For example, Fu et al. prepared a biodegradable MSNs-based nanoplatform, bMSNs-Mel@PDMC-CHP, with excellent macrophage escape, cardiac targeting, and drug release properties *via* a seed-mediated template method for the precise treatment of myocardial infarction (Fig. 5E) [80]. Briefly, they used CTAC as a mesoporous structure templating agent, TEOS as a silicon source, and TEA as a catalyst to synthesize MSNs. Hydrophobic organic solvents not only enabled the storage of the silicon source but also interacted with CTAC at the interface, assembling into oil/water emulsion micelles that served as mesoscopic templates for generating ordered mesoporous silica structures. Subsequently, a p(DMA-MPC-CD) copolymer with hydration lubrication, self-adhesive, and targeting peptide binding sites was successfully synthesized *via* free radical copolymerization. Using supramolecular host-guest interactions, adamantane-modified cardiac homing peptide (CHP) was self-assembled onto the surface of melatonin-loaded dendritic mesoporous silica nanoparticles (bMSNs). *In vivo* experiments demonstrated that bMSNs-Mel@PDMC-CHP, fabricated using CTAC *via* the template method, effectively alleviated cardiomyocyte apoptosis, improved myocardial interstitial fibrosis, and enhanced cardiac function.

Apart from CTAC, CTAB can also serve as a mesoporous structure-directing agent for MSNs. For example, the superlubricated Poly(potassium 3-sulfopropyl methacrylate)-grafted mesoporous silica nanoparticles (MSNs-NH_2_@PSPMK) were designed by Zhang et al. utilizing CTAB as a pore-forming template for the precise treatment of osteoarthritis (Fig. 5F) [81]. The grafted PSPMK polymers endowed the nanoparticles with enhanced lubricity due to the establishment of a robust hydration layer around the negative charges, while the ample mesoporous channels of MSNs enable efficient drug loading and release characteristics. When encapsulated with the anti-inflammatory agent diclofenac sodium (DS), the superlubricating nanoparticles exhibited improved lubricity while maintaining the drug release rate through enlarging the thickness of the PSPMK layer, which was briefly accomplished by arranging the precursor monomer concentration during photopolymerization. *In vitro* and *in vivo* experimental revealed that the fabricated MSNs-NH_2_@PSPMK nanoparticles *via* the template method effectively efficiently preserved the chondrocytes from degeneration, thereby suppressing the progression of osteoarthritis. Although this study proposed a synergistic effect of “hydration lubrication” and “anti-inflammatory drug release,” the underlying molecular mechanisms remain insufficiently explored. For instance, the interaction mechanism between the PSPMK polymer hydration layer and the glycosaminoglycans on the cartilage surface has not been clearly elucidated; the specific regulatory effects on chondrocyte metabolism after drug release have yet to be systematically described; and the potential impact on synovial inflammation in the joint has not been adequately evaluated. Overall, the mechanistic explanations provided are still relatively general and warrant further in-depth investigation.

In summary, the three conventional preparation methods-sol-gel, hydrothermal, and template-assisted-each offer distinct advantages and limitations for fabricating the basic MSN structure (Table 2). While these strategies provide the fundamental scaffolds, the resulting nanoparticles are merely passive carriers. To unlock their full theranostic potential by transforming them into intelligent agents that can navigate complex biological environments, precise surface engineering is paramount. Therefore, the following section will shift focus from the synthesis of the core to the functionalization of the surface, exploring the key modification strategies that serve as the fundamental 'building blocks' for advanced nanomedicine design. We will detail the underlying mechanisms for achieving the targeted, responsive, and biomimetic properties required for the advanced biomedical applications discussed later in this review.

**Table 2:** Comparison of three preparation strategies for mesoporous silica materials.

Strategies	Size Control	Morphology Control	Pore Structure Control	Large-scale Production
Sol-gel Method	Moderate	Diverse, relatively good	Generally less ordered	Simple operation, low cost
Hydrothermal Method	Precise, uniform	Easily tunable, diverse	Ordered, high crystallinity	Complex process, easy to scale up
Template Method	Precise, highly repeatable	Designable, diverse	Highly ordered, customizable	Complex process, high cost, hard to scale up

## 3 Surface-functionalized modification of MSNs

The surface characteristics of ordered mesoporous silica can be modified by introducing inorganic species or grafting various organic functional groups. Surface functionalization methods generally involve surface grafting modification and co-condensation of functional silanes on the surface of MSNs [107,108]. Over the past 50 years, the field of silica surface modification has developed significantly. It not only enables the functional modification of ordered mesoporous silica but also allows for the customization of materials for chromatographic applications [[109], [110], [111]]. In this section, we introduce several key functionalization methods for MSNs, including targeted modification, biomimetic modification, stimuli-responsive modification, and aptamer-based surface engineering. It is crucial to recognize that these strategies are not merely a collection of techniques but represent distinct philosophies for interfacing nanoparticles with complex biological systems. To illustrate how these surface engineering strategies translate into practical biomedical applications, we have summarized representative MSN-based delivery systems in Table 3. This table compares their target indications, particle sizes, surface modifications, and observed *in vivo* outcomes, providing a functional overview of how surface design influences therapeutic performance. To provide a clear and critical comparison that will serve as a roadmap for this section, we have summarized the core attributes of each approach in Table 4. This table juxtaposes their underlying mechanisms, key advantages, inherent limitations, and ideal application scenarios, offering a comprehensive framework for selecting the most appropriate strategy for a given biomedical challenge. The following subsections will delve into the details of each strategy, substantiated with recent groundbreaking examples.

**Table 3:** Summary of functional modifications of MSN-Based nanomaterials by Application, size, surface engineering, and in vivo outcome.

Nanomaterials	Target Application	Size (nm)	Surface Modification	In Vivo Outcome	Ref.
SL@M@Arg-MSNs@BA	Acute Pancreatitis	∼100–150	DSPE-PEG-SLIGRL (PAC-targeting peptide)	Restored pancreatic function; Improved survival rate	[112]
MSN-PEG-Ab-TAT	Breast Cancer (NF-κB)	∼100	p65 antibody; TAT peptide	Tumor suppression in 4T1 mice	[113]
Mito(T)-pep-Nuc(T)	Liver Metastases	∼120	Triphenyl phosphonium; NLS peptide	Synergistic PDT/PTT tumor elimination	[114]
DMSN-Au-Fe_3_O_4_	Tumor Catalytic Therapy	∼80	PEG; Au/Fe_3_O_4_ nanozymes	Tumor necrosis; high biocompatibility	[115]
ESC-HCM-B	Diabetic Nephropathy	∼150–200	Biomimetic composite membrane	Podocyte targeting; Decreased urinary protein; Improved kidney function	[116]
MSNs-NH_2_@PMPC	Osteoarthritis	∼100–120	PMPC polymer grafting	Cartilage protection; Decreased degeneration	[117]
DOX@MSN-WS_2_-HP	Tumor Chemotherapy/PTT	∼100	Benzoic-imine bond	Tumor suppression; selective cytotoxicity	[118]
MSN@DA	Anti-angiogenesis	∼100	Boronic ester bond	Tumor vessel normalization; Improved Dox efficacy	[119]
MSN_ConA_	Osteosarcoma	∼100	Acetal linker	Enhanced cytotoxicity vs free drug; selective tumor targeting	[120]
PMS/PC	Diabetic Osteopathy	∼120	Phenyl sulfide group	Improved bone formation; Decreased vascular oxidative stress	[121]
MSN@TheraVac	Cancer Immunotherapy	∼150	Diselenide bond	Improved T-cell activation; Decreased immunosuppression	[122]
Ar-MSNs-TK-PEG	Retinal Diseases	∼100	Thioketal (TK); PEG	Retinal targeting; Decreased neovascularization	[123]
DNA-MSN (DNAM)	Osteoporosis	∼100–120	AptScl56 DNA aptamer	Restored bone mass; Improved mechanical strength	[124]
InCasApt	miRNA-responsive PDT	∼150	RNA aptamer precursor; CRISPR-Cas13a	Decreased tumor migration; Improved BRG1 expression	[125]
MSN-AP	Lung Cancer Exosome Removal	∼100	EGFR-targeting aptamer	Efficient exosome elimination *via* hepatobiliary pathway	[126]

**Table 4:** Comparative analysis of surface modification strategies for MSNs.

Strategy	Underlying Mechanism	Key Advantages	Key Limitations	Ideal Application Scenarios
Targeted	Specific ligand-receptor binding	High specificity; enhanced cellular uptake	High cost; potential immunogenicity; steric hindrance	Therapy for diseases with well-defined surface biomarkers (*e.g.*, specific cancers)
Biomimetic	Camouflaging with natural cell membranes (*e.g.*, RBC, platelet)	Excellent biocompatibility; prolonged circulation; immune evasion; inherent targeting	Complex fabrication; potential for batch-to-batch variability; reduced drug loading space	Systemic drug delivery requiring long circulation times and avoidance of the RES
Stimuli-Responsive	Cleavage of specific chemical bonds in response to microenvironmental cues (pH, ROS, etc.)	"On-demand" drug release; reduced premature leakage; improved therapeutic index	Potential for incomplete response; sensitivity may not perfectly match biological triggers	Tumor therapy (acidic & high-ROS TME); inflammation-targeted therapy
Aptamer-Based	3D structural recognition of targets by nucleic acid aptamers	High specificity & affinity; low immunogenicity; easy synthesis; versatile targets	Susceptibility to nucleases *in vivo*; smaller size may lead to rapid renal clearance	Targeted drug delivery; biosensing; theranostics combining targeting and diagnostics

### 3.1 Targeted modification

Nanoparticles are capable of enhancing bioavailability and decreasing the necessary therapeutic dose at the target site via targeted delivery and controlled or selective drug release. Mechanistically, this is achieved by conjugating specific ligands (*e.g.*, antibodies, aptamers) to the MSN surface. This functionalization converts the nanoparticle's non-specific biodistribution into a highly specific interaction with target cell receptors, resulting in enhanced cellular uptake at the disease site. As a consequence, tumor targeting efficacy is significantly improved, which allows for a higher local drug concentration and a more potent therapeutic outcome with reduced systemic side effects. This effectively overcomes multidrug resistance [127,128]. Among various nanoparticles, MSNs have been intensively investigated in the past two decades, especially for their promising potential in targeted drug delivery [129]. By means of targeted delivery and controlled drug release, MSNs can remarkably improve drug utilization at the target site. Consequently, the required dosage is reduced, demonstrating significant clinical value.

**Fig. 6:**
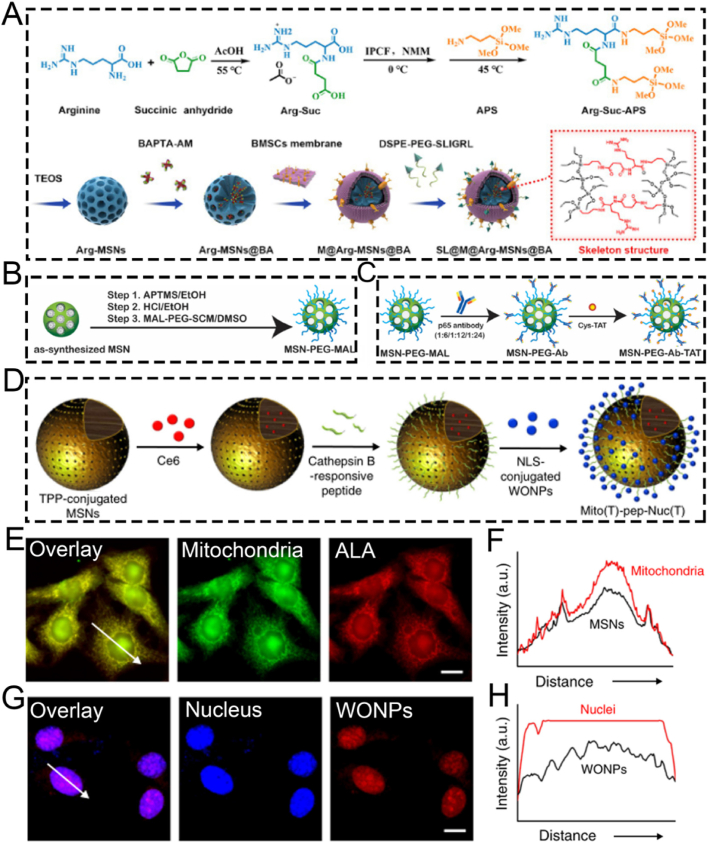
(A) Schematic illustration of the SL@Arg-MSNs@BA preparation. (B) Schematic illustration of the MSN synthesis using a heterobifunctional PEG crosslinker. (C) The MAL terminus of MSN-PEG reacts with the sulfhydryl groups of the antibody and the Cys-TAT peptide. (D) Schematic illustration of the nucleus and mitochondria dual-targeted nanoplatform Mito(T)-pep-Nuc(T). (E) Fluorescence confocal images of mitochondrial targeting and (F) corresponding analysis. (G) Fluorescence confocal images of nuclear targeting and (H) corresponding analysis. (A) Reproduced with permission [112]. Copyright 2024, American Chemical Society. (B, C) Reproduced with permission [113]. Copyright 2020, Elsevier. (D–H) Reproduced with permission [114]. Copyright 2019, Springer Nature.

For example, He and colleagues successfully proposed an organically bridged, trypsin-responsive MSN matrix, SL@M@Arg-MSNs@BA, which was functionalized with pancreatic acinar cell (PAC)-targeting peptides SLIGRL. This matrix was designed to specifically target PACs for the precise treatment of acute pancreatitis (AP) (Fig. 6A) [112]. Specifically, the mesenchymal stem cell membrane layer and the surface functionalization with PAC-targeting ligands DSPE-PEG-SLIGRL enabled MSNs to recruit inflammatory cells and accurately target PACs. As a result, there was a peak distribution in the pancreas at 3 h, and the accumulation was 4.7 times higher compared to bare MSNs. After the skeleton of the bioinspired MSNs was degraded by over-activated trypsin, BAPTA-AM was released on demand in damaged PACs. This effectively removed the intracellular calcium overload, restored cellular redox homeostasis, halted the inflammatory cascade, and inhibited cell necrosis by suppressing the CaMK-Ⅱ/p-RIP 3/pMLKL/caspase-8,9 and IκBα/NF-κB α/TNF-α/IL-6 signaling pathways. *In vivo* experiments showed that SL@M@Arg-MSNs@BA significantly restored pancreatic function, decreased lipase and amylase levels, and increased the survival rate of mice. In conclusion, the specifically targeted SL@M@Arg-MSNs@BA developed in this study represents a potentially promising strategy using PAC-targeting peptides for the clinical application of AP treatment. Nevertheless, this nanoplatform has so far only been validated for loading the calcium chelator BAPTA-AM, and its applicability to other anti-arthritic drugs remains unverified. Considering the significant differences in physicochemical properties (such as molecular weight and hydrophilicity/hydrophobicity) among various drugs, these factors may affect drug loading efficiency, release kinetics, and synergistic therapeutic effects. Therefore, validation with a single drug is insufficient to fully demonstrate the versatility and adaptability of this platform, and further studies are needed to evaluate its broader applicability.

The transcription factor NF-κB (p65/p50) is recruited to the cytoplasm by its inhibitor IκBα. Once activated, the Rel proteins p65/p50 are released from IκBα and translocated through nuclear pores. This process regulates the expression of numerous genes and makes NF-κB a potential target for cancer therapy. In the study by Mou et al., the NF-κB-targeting nanoparticle/antibody complex MSN-PEG-Ab-TAT, which consists of MSN, the TAT peptide, and a p65-specific antibody, was employed to capture the perinuclear domain Rel protein p65. This disrupted its translocation near the nuclear pore (Fig. 6B and C) [113]. The surface modification of MSNs with TAT peptides promotes non-endocytic cell membrane transduction and facilitates their accumulation in the perinuclear region. In this region, p65-specific antibodies can effectively target and capture active NF-κB p65. Furthermore, the combined treatment of mice with MSNs and Dox demonstrated significant therapeutic efficacy in 4T1 tumor-bearing mice. The novel therapeutic approach using antibody-surface-modified MSNs, which takes advantage of the “nuclear focusing” and “size-exclusion blocking” effects to target transcription factors, is versatile and can be adapted to regulate other transcription factors.

In contrast to targeting a single agent, surface-modified MSNs capable of dual-substance targeting can further boost targeting efficiency, significantly minimizing the side effects of therapeutic agents. For example, Hu and colleagues developed a nanocomplex, Mito(T)-pep-Nuc(T), which contains W18O49 nanoparticles and MSNs. This nanocomplex can simultaneously target the nucleus and mitochondria *via* triphenyl phosphonium and the nuclear localization sequence, respectively, for eradicating unresectable liver metastases (Fig. 6D) [114]. Briefly, the nuclear-gated tungsten oxide nanoparticles (WONP) were conjugated to mitochondria-specific MSNs containing the photosensitizer Ce6 through cathepsin B-cleavable peptides. Upon laser irradiation, the reactive oxygen species (ROS) generated by hepatocytes were scavenged by WONP, leading to the loss of its photothermal heating ability and protecting hepatocytes from laser-induced thermal damage. In hepatic metastatic cancer, the cleavable peptide linker allowed WONPs and MSNs to separately target the nucleus and mitochondria (Fig. 6E–H), enabling synergistic photodynamic and photothermal therapy for cancer elimination.

In summary, targeted modification strategies, whether employing antibodies, peptides, or other ligands, fundamentally rely on specific ligand-receptor interactions to achieve active targeting. Antibody-based targeting offers unparalleled specificity but is often hindered by high cost, large size, and potential immunogenicity. In contrast, peptide-based targeting provides a smaller, more cost-effective alternative with better tissue penetration, though its binding affinity may be lower. The choice of strategy is therefore highly dependent on the application scenario: for diseases with highly specific and well-characterized biomarkers (*e.g.*, HER2+ cancer), antibodies are superior; for broader applications requiring deep tissue access, peptides may be more pragmatic. A key challenge remains in balancing targeting efficiency with off-target effects and systemic clearance.

### 3.2 Biomimetic modification

Despite the substantial progress in nanomaterials over the past decade, the biocompatibility of materials remains a key concern for researchers. This is because it determines the materials' capacity to adapt to host responses *in vivo* [[130], [131], [132]]. Biomaterials used for vascular grafts or stents usually necessitate cytotoxicity evaluation *via* cell viability assays and hemocompatibility tests. Further to this, biodegradability is of particular importance for special materials like absorbable stents, as these are engineered to offer only temporary functionality [133]. Significantly, the biocompatibility of various nanomaterials can be regulated through biomimetic surface modifications [[134], [135], [136], [137]]. These modifications fundamentally alter the nanoparticle's surface chemistry and zeta potential, which directly governs its interaction with blood opsonins and immune cells. A successful modification can transform a recognizable foreign particle into a “self-like" entity, thereby modulating the immune response, prolonging circulation half-life, and ultimately dictating its biodistribution. The past decade has witnessed the extensive applications of inorganic nanoparticles with outstanding biocompatibility in emerging nanocatalytic tumor therapy [[138], [139], [140], [141], [142], [143]].

**Fig. 7:**
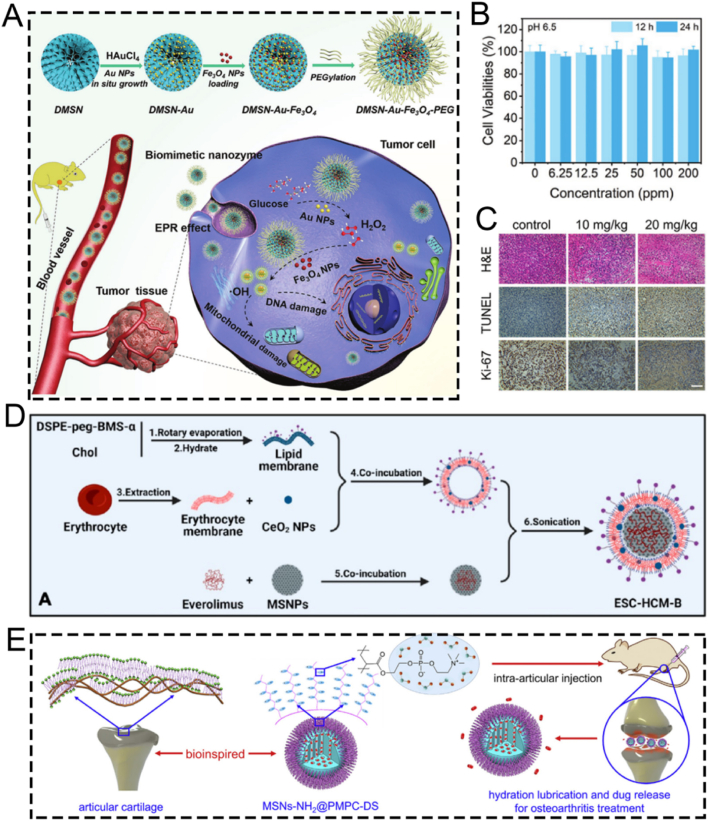
(A) Schematic diagram of nanocatalytic oncology therapy with “non-toxic drugs” *via* biomimetic inorganic nanomedicine-activated cascade catalytic reaction. (B) Relative viability in 4T1 cells after incubation with different concentrations of DMSN-Au-Fe_3_O_4_. (C) H&E staining, TUNEL staining to observe pathological alterations and Ki-67 immunohistochemical staining to observe cell proliferation of tumor tissues in each group after 15 days treatment period. Scale bar: 100 μm. (D) Schematic diagram of ESC-HCM-B preparation. (E) Schematic illustration of the design of superlubricated nanospheres. (A–C) Reproduced with permission [115]. Copyright 2019, Wiley. (D) Reproduced with permission [116]. Copyright 2023, American Chemical Society. (E) Reproduced with permission [117]. Copyright 2020, Elsevier.

For instance, the outstanding biocompatibility of MSN nanocomposites can be achieved through surface modification with PEG. Shi et al. developed a biomimetic PEG-modified MSN-based nanoplatform, DMSN-Au-Fe_3_O_4_, which consists of ultrasmall Au and Fe_3_O_4_ NPs. This was done to enhance biocompatibility and physiological stability for nanocatalytic tumor therapy (Fig. 7A) [115]. The Au NPs emulated glucose oxidase, while the Fe_3_O_4_ NPs mimicked peroxidase. The Au NPs selectively oxidized β-D-glucose into gluconic acid and hydrogen peroxide (H_2_O_2_). Subsequently, the Fe_3_O_4_ NPs catalyzed this H_2_O_2_ to generate ROS, thereby inducing tumor cell death. Importantly, cytotoxicity assays verified the high biocompatibility and biosafety of the nanoplatform (Fig. 7B). *In vivo* hematoxylin and eosin (H&E) and terminal deoxynucleotidyl transferase dUTP nick end labeling (TUNEL) staining assays showed significant tumor cell damage and necrosis in the group treated with the nanoplatform, demonstrating the excellent therapeutic efficacy of the biomimetic PEG-modified nanoplatform (Fig. 7C). Overall, this research presents a rational design for a biomimetic PEG-modified MSN-based nanoplatform, enabling efficient and safe nanocatalytic tumor therapy. Even though, DMSN-Au-Fe_3_O_4_ nanoplatform primarily relies on the EPR effect of tumor vasculature to achieve passive targeting, without the incorporation of active targeting ligands. As a result, its targeting efficiency may be relatively limited. Further studies introducing active targeting strategies could potentially improve the tumor accumulation and therapeutic efficacy of such nanoplatforms.

PEG modification improves the biocompatibility of MSNs, biomimetic composite membrane modification is also an excellent alternative. For example, Zeng et al. reported a multifunctional MSNs-based nanocomposite, ESC-HCM-B, modified with an enhanced biohomogeneous composite membrane. This nanocomposite is designed for the targeted release of ROS inhibitors and mTOR to enhance biocompatibility for the synergistic treatment of diabetic nephropathy (DN) (Fig. 7D) [116]. MSNs endow the ESC-HCM-B nanocomposite with a high drug-loading capacity. Due to the presence of the biomimetic composite membrane, ESC-HCM-B can avoid immune phagocytosis, enable self-valve-controlled drug release, and maintain high stability. *In vitro*, ESC-HCM-B demonstrates excellent podocyte-targeting efficiency, without significant cytotoxicity or pro-apoptotic effects. In *in vivo* experiments, ESC-HCM-B can specifically target the glomerular and podocyte regions of the kidney. Additionally, ESC-HCM-B reduces high glucose-induced ROS levels in podocytes and restores mitochondrial energy metabolism. Importantly, ESC-HCM-B significantly reduces urinary protein levels in DN rats and delays glomerulosclerosis. Overall, this biomimetic MSN-based nanocomposite, through surface modification with a biomimetic composite membrane, offers a safe and effective strategy for DN treatment.

Beyond their applications in tumor and DN treatments, surface-modified biomimetic MSNs are also employed in the treatment of osteoarthritis. Inspired by the structure of phosphatidylcholine lipids, a typical constituent of the cartilage matrix, Zhang and colleagues fabricated superlubricating poly(2-methacryloyloxyethyl phosphorylcholine)-modified nanopolymers, namely MSNs-NH_2_@PMPC, *via* photopolymerization. This was done to enhance biocompatibility for the efficient treatment of osteoarthritis (Fig. 7E) [117]. The surface-functionalized modification with poly(2-methacryloyloxyethyl phosphorylcholine) imparted excellent lubrication properties to MSNs-NH_2_@PMPC. MSNs-NH_2_@PMPC improved lubrication due to the formation of a robust hydration layer around the zwitterionic charges of the nanopolymers. Meanwhile, the use of MSNs enabled the targeted delivery of anti-inflammatory drugs. Experimental findings indicated that the superlubricating drug-loaded nanopolymers impeded the progression of osteoarthritis by increasing cartilage anabolic components and suppressing pain-related genes and catabolic proteases. The fabricated nanopolymers MSNs-NH_2_@PMPC, featuring excellent lubrication and sustained drug delivery capabilities, show great potential as an effective intra-articular nanomedicine for the treatment of osteoarthritis.

Collectively, the biomimetic modifications discussed highlight two core design principles: “stealthing" and “camouflaging." PEGylation (*e.g.*, DMSN-Au-Fe_3_O_4_) exemplifies the “stealth" approach, creating a hydrophilic layer to passively evade immune surveillance. In contrast, cell membrane coating (*e.g.*, ESC-HCM-B) and matrix-mimicking polymers (*e.g.*, MSNs-NH_2_@PMPC) represent “camouflage," actively mimicking endogenous structures to achieve superior biocompatibility and, in some cases, homologous targeting. The choice of strategy depends on the desired balance between simplicity (PEGylation) and functional complexity (membrane coating), with the latter offering more sophisticated biological interactions at the cost of a more complex fabrication process.

### 3.3 Stimuli-responsive strategies for surface modification

The open mesopores of MSNs facilitate the relatively easy loading of therapeutic agents into their pores. However, these agents can freely diffuse out through the same routes, significantly diminishing their efficacy and potentially exacerbating certain side effects. Thus, it is crucial to devise various strategies to close the pore entrances and prevent the unintended release of the cargo. Over the past several years, numerous strategies have been developed for MSN-based smart nanocarriers to seal the pore gates, enabling the on-demand release of therapeutic agents in response to specific stimuli. In this subsection, we elaborate in detail on the fashionable pH-responsive and ROS-responsive strategies for the surface modification of MSNs.

#### 3.3.1 pH-responsive strategies

The pH-stimulated system is a standard stimulus-responsive drug delivery platform, especially suitable for tumor-targeted release. The tumor microenvironment is weakly acidic (pH 5.5–7.0), which can act as a stimulus for the targeted release of drugs from carriers into tumors [144,145].

**Fig. 8:**
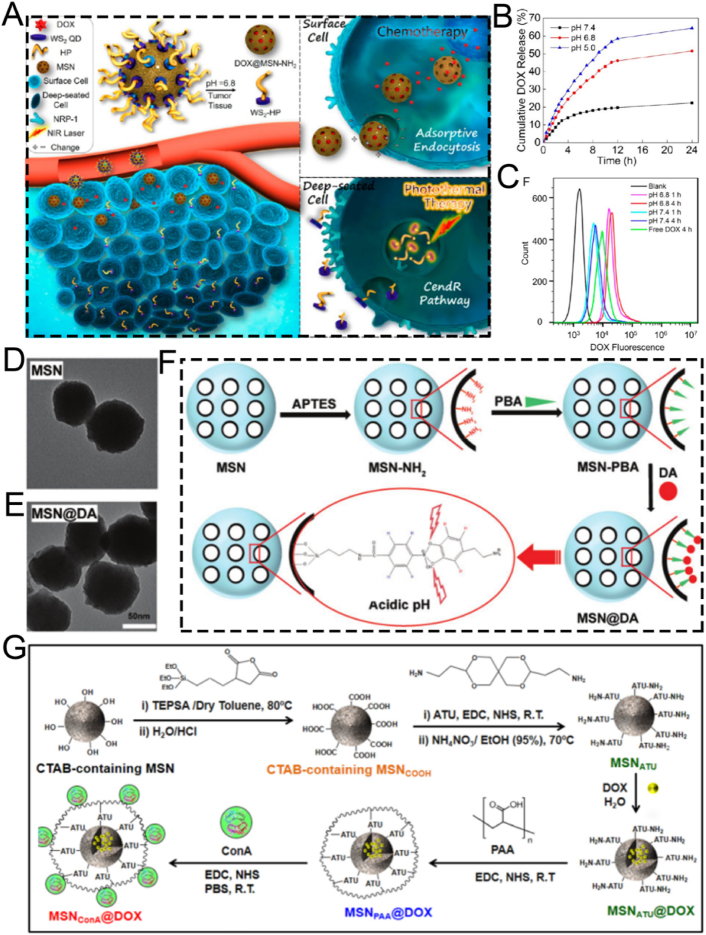
(A) Schematic illustration of the preparation and application of DOX@MSN-NH_2_. (B) Cumulative DOX release profiles of DOX@MSN-WS_2_-HP at different pH conditions. (C) Flow cytometry analysis of cells after various treatments. (D) Representative TEM images of MSN. (E) Representative TEM images of MSN@DA. (F) Schematic illustration of MSN@DA synthesis. (G) Schematic synthesis of pH-responsive MSN_ATU_@DOX nanoplatforms. (A–C) Reproduced with permission [118]. Copyright 2017, American Chemical Society. (D–F) Reproduced with permission [119]. Copyright 2019, Wiley. (G) Reproduced with permission [120]. Copyright 2017, Elsevier.

Zhang et al. engineered a multifunctional nanosystem based on MSNs modified with tungsten disulfide quantum dots (WS_2_-HP), namely DOX@MSN-WS_2_-HP. This nanosystem was functionalized *via* a benzoic-imine bond to achieve pH responsiveness. It functioned as a pH-responsive “cluster bomb” and was conjugated with the tumor-homing/penetrating peptide tLyP-1 for efficient tumor suppression (Fig. 8A) [118]. The pH-responsive benzoic-imine bond connected the “cluster bomb” and the “distributor,” remaining stable under normal physiological conditions. Nevertheless, upon reaching the weakly acidic tumor microenvironment, the nanosystem dissociated into two components in a responsive manner. One component was DOX@MSN-NH_2_, which enabled effective chemotherapy for tumors. The other component was WS_2_-HP, which demonstrated excellent tumor-penetrating ability for near-infrared (NIR) light-activated PTT in deep tumor cells (Fig. 8B and C). Experimental results indicated that the pH-responsive DOX@MSN-WS_2_-HP, achieved through benzoic-imine bonds, exhibited specific cytotoxicity against tumor cells, demonstrating significant antitumor effects and holding promising potential for clinical applications.

Borate ester bonds, together with benzoic-imine bonds, act as pH-responsive chemical linkages that are crucial in smart nanomedicine delivery systems, facilitating the specific release of therapeutic agents. To achieve the responsive delivery of the anti-angiogenic drug dopamine, Nie and colleagues synthesized an MSN-based Dox-loaded nanoplatform, MSN@DA, which enables the pH-responsive release of dopamine through the breakage of boronic ester bonds to enhance the anti-tumor effect (Fig. 8D–F) [119]. Specifically, MSNs were functionalized with amine-conjugated phenylboronic acid molecules, allowing dopamine to be loaded *via* the formation of boronate esters. Under weakly acidic conditions, the boronate ester bonds between dopamine and phenylboronic acid on MSNs undergo hydrolysis, enabling precisely controlled dopamine release. This smart delivery system significantly inhibits the migration and tube formation of vascular endothelial cells. Loading dopamine into functionalized MSNs not only significantly extends the circulation half-life of this small molecule but also induces tumor vessel normalization, thereby enhancing the chemotherapeutic efficacy of Dox. This research indicates that pH-responsive MSNs show great potential for *in vivo* dopamine delivery. The tumor vessel normalization effect induced by dopamine represents a promising adjuvant strategy for cancer chemotherapy.

Apart from benzoic-imine bonds and borate ester bonds, the acetal linker is frequently utilized as a pH-responsive modification component to further boost the efficiency of diagnostic and therapeutic applications. For example, Vallet-Regí et al. have successfully developed a pH-responsive, acetal linker-modified, versatile MSN-based nanoplatform, MSN_ConA_, for Dox loading for the treatment of osteosarcoma [120]. This nanoplatform consists of two crucial components: a pH-responsive acetal linker for precise drug delivery and a targeting ligand for specific recognition and selective tumor cell targeting (Fig. 8G). When compared with healthy pre-osteoblastic cells (MC3T3-E1), this multifunctional nanoplatform exhibits significantly enhanced internalization efficiency in SA-overexpressing human osteosarcoma cells. Their results indicated that the nanoplatform demonstrated an 8-fold higher cytotoxicity towards tumor cells compared to free drugs, leading to nearly 100 % death of osteosarcoma cells. The synergistic integration of different structural components has enabled the construction of a unique nanoplatform that not only enhances the antitumor efficacy but also reduces the toxicity to normal cells. These findings strongly support the crucial role of pH-responsive MSN-based nanoplatforms in the targeted therapy of osteosarcoma.

#### 3.3.2 ROS-responsive strategies

ROS, encompassing H_2_O_2_, singlet oxygen (^1^O_2_), hydroxyl radicals (•OH), and superoxide anions (O_2_^•-^), typically exhibit lower concentrations in normal tissues when contrasted with the tumor microenvironment (TME), inflamed tissues, or other damaged areas [[146], [147], [148]]. Consequently, researchers have engineered diverse ROS-responsive nanomaterials for the diagnosis and treatment of diseases like tumors and inflammation.

**Fig. 9:**
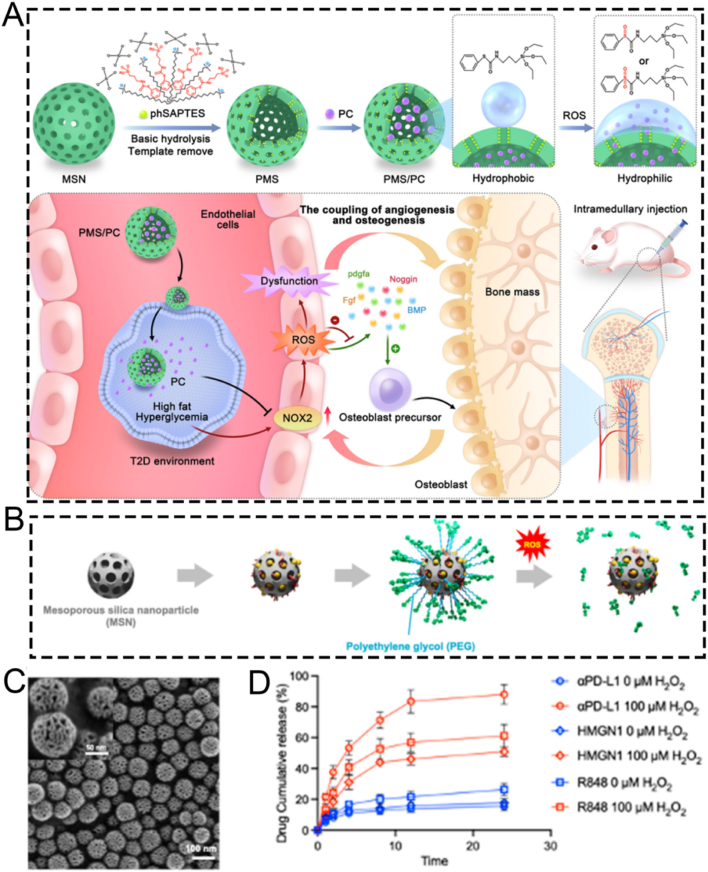
(A) Schematic diagram of the ROS-responsive PMS/PC delivery system, its function and potential mechanisms. (B) Schematic illustration of MSN@TheraVac synthesis. (C) Representative scanning electron microscope image of MSN. (D) Cumulative release profile of various drugs at different pH conditions. (A) Reproduced with permission [121]. Copyright 2022, Elsevier. (B–D) Reproduced with permission [122]. Copyright 2023, American Chemical Society.

For example, the excessive accumulation of ROS elicits oxidative stress responses in patients with long-term diabetes. This results in bone fragility and an elevated risk of fracture. Oxidative stress is a characteristic trait of diabetes mellitus, playing a pivotal role in disease progression, the development of complications, and the emergence of drug resistance. To enhance the treatment of diabetes, Wang's research team pioneered the fabrication of an innovative ROS-responsive drug delivery nanoplatform, PMS/PC. This nanoplatform utilizes phenyl sulfide mesoporous silica nanoparticle (PMS) as a matrix for encapsulating proanthocyanidin (PC), thereby establishing a potential therapeutic approach for promoting bone formation under diabetic conditions (Fig. 9A) [121]. The synthesized spherical PMS particles exhibited a highly ordered two-dimensional hexagonal mesoporous structure, which significantly augmented their loading capacity for PC molecules. The wettability of the inner surface of the nanopores in PMS could be effectively regulated through surface modification with hydrophobic PhS groups.

Also, the PhS groups endow PMS/PC with ROS responsiveness. Specifically, under ROS stimulation, the hydrophobic PhS groups can be oxidized into hydrophilic phenyl sulfoxide or phenyl sulfone groups featuring electron-withdrawing properties. This transition in wettability effectively modulates the surface properties of the nanopores, thereby triggering the release of the loaded PC. Subsequently, the intelligent drug release performance of the PMS/PC nanoplatform under ROS stimulation was systematically investigated in experiments. The experimental findings revealed that the responsive release of PC from the ROS-sensitive PMS/PC delivery system, combined with its dual functions of eliminating excessive ROS and regulating ROS levels, effectively maintains intracellular ROS homeostasis. PMS/PC effectively mitigates vascular oxidative stress by suppressing the excessive generation of ROS, promoting the synergistic coupling between angiogenesis and osteogenesis. This significantly enhances osteoblast differentiation *in vitro* and substantially improves bone formation capacity *in vivo*. In summary, the ROS-responsive PMS/PC nanoplatform exhibits remarkable therapeutic potential for the treatment of diabetic osteopathy, holding promising application prospects in suppressing oxidative stress induced by excessive ROS.

MSNs have emerged as one of the most promising nanocarriers in drug delivery systems. This is attributed to their outstanding biocompatibility, tunable size characteristics, straightforward synthesis process, and low production costs [149,150]. However, conventional MSNs face significant limitations in peptide or protein delivery due to their restricted pore sizes and insufficient biodegradation kinetics. To address this challenge, Yang et al. engineered a novel type of large-pore MSNs, namely MSN@TheraVac, with ROS-responsive properties. These were specifically designed for the efficient delivery of therapeutic vaccines (TheraVac), a potent therapeutic cancer vaccine. TheraVac consists of HMGN1, R848, and αPD-L1 antibodies and is used for tumor-specific immunity treatment (Fig. 9B) [122]. HMGN1 and R848 were successfully encapsulated within the enlarged mesopores of the MSNs. Meanwhile, αPD-L1 antibodies were conjugated to the nanoparticle surface *via* diselenide bonds and PEG-based crosslinkers (Fig. 9C). This innovative design facilitated rapid and precise drug release in response to the elevated ROS levels that are characteristic of the TME. This was achieved because the diselenide bonds underwent efficient cleavage under such conditions. Specifically, upon arriving at the tumor site, the αPD-L1 antibodies exhibited ROS-responsive release *via* the oxidative cleavage of diselenide bonds within the TME (Fig. 9D). This precisely regulated release mechanism enabled effective binding to PD-1-expressing cells, thereby alleviating immunosuppression and preventing the exhaustion of effector T cells. Overall, the ROS-responsive MSN nanoplatform showcases remarkable potential as an advanced cancer vaccine delivery system. It demonstrates potent antitumor efficacy while effectively overcoming the inherent limitations associated with the administration of TheraVac.

Beyond diselenide bonds functioning as ROS-responsive linkers, thioketal (TK) groups have emerged as a widely utilized and well-established class of ROS-cleavable linkages in drug delivery systems. For example, Lee et al. reported an innovative MSN-based TK-conjugated nanoplatform, Ar-MSNs-TK-PEG, which features ROS-triggered surface charge reversal. This enables efficient delivery of the humanin peptide to retinal pigment epithelial cells (ARPE-19) for the effective treatment of neovascular retinal diseases [123]. Specifically, the surface engineering strategy entailed incorporating 20 % acetyl-L-arginine (Ar) to confer partial positive charges, while conjugating 80 % TK-linked methoxy polyethylene glycol (mPEG) as an ROS-responsive shielding layer. This achieved precise control over the nanoparticle's properties. The engineered MSNs leverage their oxidation stress-responsive charge reversal properties to achieve sustained retention within the anionic vitreous network. Conversely, the study did not directly compare the Ar-MSNs-TK-PEG drug delivery system with currently used clinical therapies. Consequently, it remains unclear whether this nanoplatform offers specific advantages over existing treatments with regard to efficacy, safety, therapeutic cost, and patient compliance. This limitation hinders a comprehensive evaluation of its potential for clinical translation.

Additionally, they facilitate targeted diffusion to retinal cells upon exposure to oxidative stress conditions. These MSNs exhibited an exceptional peptide loading capacity while maintaining excellent biocompatibility, as indicated by the undetectable toxicity in both retinal cells and ocular tissues. The protective properties of MSNs functioned as an efficient barrier system. This system effectively minimized premature drug release and guaranteed the stability of the payload until the intended retinal targets were reached. Both *in vitro* and *in vivo* experimental findings indicated that this innovative strategy significantly augmented the therapeutic potential of the encapsulated drugs, presenting promising prospects for enhancing clinical treatment outcomes. In conclusion, the developed ROS-responsive nanoplatform, Ar-MSNs-TK-PEG, exhibits remarkable efficacy in alleviating oxidative stress and preventing the formation of retinal neovascularization.

As discussed, stimuli-responsive strategies utilize a variety of chemical linkers to achieve controlled release. A critical comparison reveals distinct advantages and limitations. Benzoic-imine bonds and acetal linkers are both pH-sensitive, but they respond to different pH ranges; imine bonds typically cleave in the mildly acidic tumor microenvironment (pH ∼6.5), making them ideal for extracellular release, whereas acetal linkers often require the lower pH of endo/lysosomes (pH ∼5.0) for efficient cleavage, suiting them for intracellular drug delivery. Boronic ester bonds also offer pH-responsiveness but with the unique ability to bind with diols, enabling a different mode of cargo attachment. For ROS-responsiveness, diselenide bonds are highly sensitive to the redox environment but can be less stable in circulation, while thioketal (TK) linkers are specifically cleaved by higher ROS levels found inside cancer cells, offering better stability and specificity. Therefore, the selection of a specific linker must be carefully matched to the biological barrier it is designed to overcome and the specific subcellular location targeted for drug release.

### 3.4 Strategies for surface modification using aptamers

Aptamers, a class of functional oligonucleotide molecules featuring unique three-dimensional structures, are derived via *in vitro* screening techniques, such as Systematic Evolution of Ligands by Exponential Enrichment (SELEX) [[151], [152], [153]]. These molecules are celebrated for their high specificity and potent binding affinity in the nanomolar or even picomolar range. This property endows them with the ability to precisely recognize and bind to a diverse array of biological targets, including proteins, peptides, carbohydrates, small molecule compounds, toxins, and even intact living cells [[154], [155], [156]]. In the realm of drug delivery, aptamers function as innovative targeting ligands. By specifically recognizing surface markers on target cells, they can facilitate precise drug delivery [157,158]. This not only significantly enhances the selectivity and efficacy of treatments but also mitigates toxic side effects on non-target tissues. The remarkable targeting capabilities of aptamers underscore their extensive application potential in targeted therapy, molecular diagnostics, and biosensing [[159], [160], [161]]. Sclerostin antibody has the potential to reactivate osteoblasts, augment bone mass and strength, and might serve as a specific targeted agent for the treatment of osteoporosis.

**Fig. 10:**
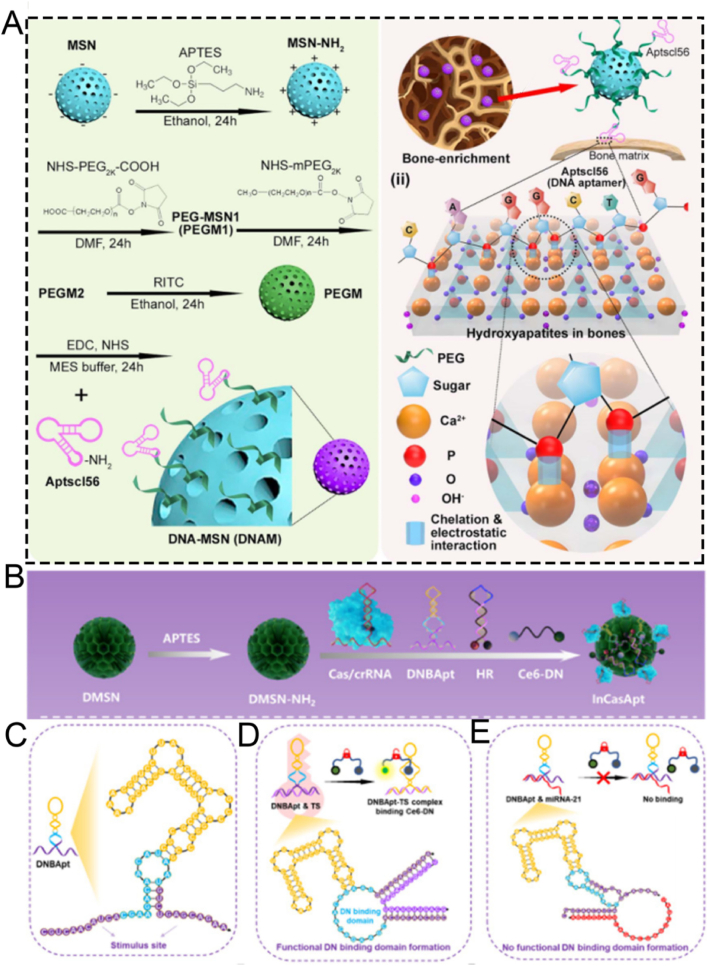
(A) Schematic illustration of the synthesis of DNAM and its application as a bone-targeting ligand. (B) Schematic diagram of aptamer theranostic platform InCasApt. (C) Cartoon on the left and secondary structure on the right of the aptamer DNBApt. (D) Binding of the DNBApt-TS complex capable of conjugating Ce6-DN (upper panel) and the secondary architecture of the DNBApt-TS complex (lower panel). (E) Impermissible Ce6-DN binding for the formation of the DNBApt-miRNA-21 compound (top) and the secondary architecture of the DNBApt-miRNA-21 compound (bottom). (A) Reproduced with permission [124]. Copyright 2022, Elsevier. (B–E) Reproduced with permission [125]. Copyright 2024, American Chemical Society.

Consequently, Zhou et al. devised a novel and facilely synthesized bone-targeting nanomedicine (DNA-MSN, DNAM), which consists of polyethylene glycol-modified dendritic MSN and a sclerostin-targeting DNA aptamer (AptScl56). This nanomedicine is designed to direct bone attachment and capture sclerostin for the precise treatment of osteoporosis (Fig. 10A) [124]. The mesoporous structure of MSN effectively protects the immobilized AptScl56 from accelerated degradation or renal filtration, thus prolonging it's *in vivo* half-life. Given the interaction between the bone calcium of hydroxyapatite and the phosphate groups of the DNA aptamer AptScl56, the DNAM-immobilized AptScl56 layer not only directly adheres to the bone tissue of ovariectomized mice but also captures sclerostin *in situ* with picomolar affinity, thereby exerting a dual therapeutic effect. Significantly, DNAM exhibited a remarkable ability to substantially reverse the elevated serum sclerostin levels, effectively restoring the osteoporosis-induced bone loss to physiological levels. It also enhances the mechanical properties of femoral bone tissue and morphological parameters, normalizes serum bone turnover markers, and shows no systemic toxicity. This study introduces a novel MSN-based nanoplatform characterized by DNA aptamer-mediated surface functionalization, presenting a promising strategy for nanomedicine-based therapeutic interventions in the treatment of osteoporosis. Nonetheless, this study employed only the ovariectomized mouse model to simulate postmenopausal osteoporosis, without incorporating other types of osteoporosis models. Given the substantial differences in pathological mechanisms among various forms of osteoporosis, validation in a single model may not fully capture the therapeutic applicability and universality of this nanoparticle across different etiologies. Therefore, further studies utilizing a broader range of osteoporosis models are warranted to comprehensively evaluate the versatility and translational potential of this nanoplatform.

Beyond DNA aptamers, there is an urgent and immediate requirement for the development of an engineered RNA aptamer device. This device should be able to detect a wide variety of biomarkers, which is essential for accurately assessing pathological conditions and optimizing treatment strategies. For instance, Dong et al. designed an MSN-based integrated nano CRISPR-Cas13a/RNA aptamer theranostic platform named InCasApt. This platform comprises a Cas13a/crRNA complex, a hairpin reporter molecule, a dinitroaniline-caged Ce6 photosensitizer (Ce6-DN), and a DN-bound RNA aptamer precursor (DNBApt). The InCasApt platform was developed to achieve miRNA-responsive PDT, enabling simultaneous biomarker detection and biomarker-mediated therapy (Fig. 10B) [125]. In normal cells, the nano InCasApt system remained in an inactive state. In contrast, the increased concentration of miRNA-155 in tumor cells activates the trans-cleavage function of Cas13a (Fig. 10C). This enzymatic activity led to the cleavage of the hairpin reporter gene. Consequently, the transduction sequence (TS) tagged with a quencher dye was liberated. Upon its release, the TS sequence folded and then bound to the stimulation site of DNBApt. Subsequently, Ce6-DN attached to the DN domain of DNBApt. This attachment restored the photosensitive characteristics of the system. The restoration of these properties promoted the generation of ROS, ultimately enabling the effective implementation of PDT (Fig. 10D). Simultaneously, this mechanism inhibited tumor migration by downregulating the activities of miRNA-155 and miRNA-21, and concurrently enhanced the expression of the tumor suppressor gene BRG1 (Fig. 10E). These findings highlight the multifunctional utility of the MSN-based RNA aptamer theranostic platform InCasApt as a targeted approach for miRNA intervention. This presents new opportunities for the seamless integration of diagnostic and therapeutic strategies.

MSNs, acknowledged as a relatively safe biomaterial, are rapidly absorbed by the liver and subsequently promptly excreted into the gastrointestinal tract [162,163]. MSNs meet the basic requirements for serving as a delivery vehicle, facilitating the transport of attached exosomes from the bloodstream to the gastrointestinal tract. In a study conducted by Jia et al., MSNs were chemically functionalized with epidermal growth factor receptor (EGFR)-targeting aptamers (MSN-AP) to enable precise recognition and binding of exosomes secreted by A549 human lung cancer cells, which are characterized by high EGFR expression (A-Exo) [126,164,165]. The molecular interaction between MSN-AP and A-Exo, involving both specific recognition and electrostatic forces, enabled the efficient elimination of A-Exo through the hepatobiliary pathway. The aptamer-functionalized nanoparticles proposed in this research offer an innovative strategy for removing carcinogenic exosomes from the bloodstream to the small intestine, thus establishing a novel mechanism for expelling harmful circulating biological agents from the body.

Having detailed the fundamental 'toolkit' of surface modification strategies in the preceding sections-from targeting and biomimetics to stimuli-responsive and aptamer-based engineering-it becomes clear that the true power of MSN-based nanomedicine lies not in applying these tools in isolation, but in their synergistic integration. The following sections will therefore transition from discussing these foundational strategies as individual components to showcasing how they are combined into cohesive, multifunctional nanoplatforms to address specific challenges in diagnostics (Chapter 4) and therapeutics (Chapter 5), defining the forefront of MSN-based nanomedicine.

## 4 Diagnostic applications of MSN-based nanoplatforms

### 4.1 Biosensors

The remarkable specific surface area and excellent pore connectivity of MSNs make them ideal carriers for encapsulating guest molecules. The unique microstructure of MSNs improves the accessibility and subsequent chemical interactions between the encapsulated substances and external agents, thereby attaining enhanced sensitivity and specificity. As a result, nanoplatforms based on MSNs have also been widely applied in analytical chemistry, acting as crucial tools for the qualitative and quantitative analysis of specific biochemical entities [166].

**Fig. 11:**
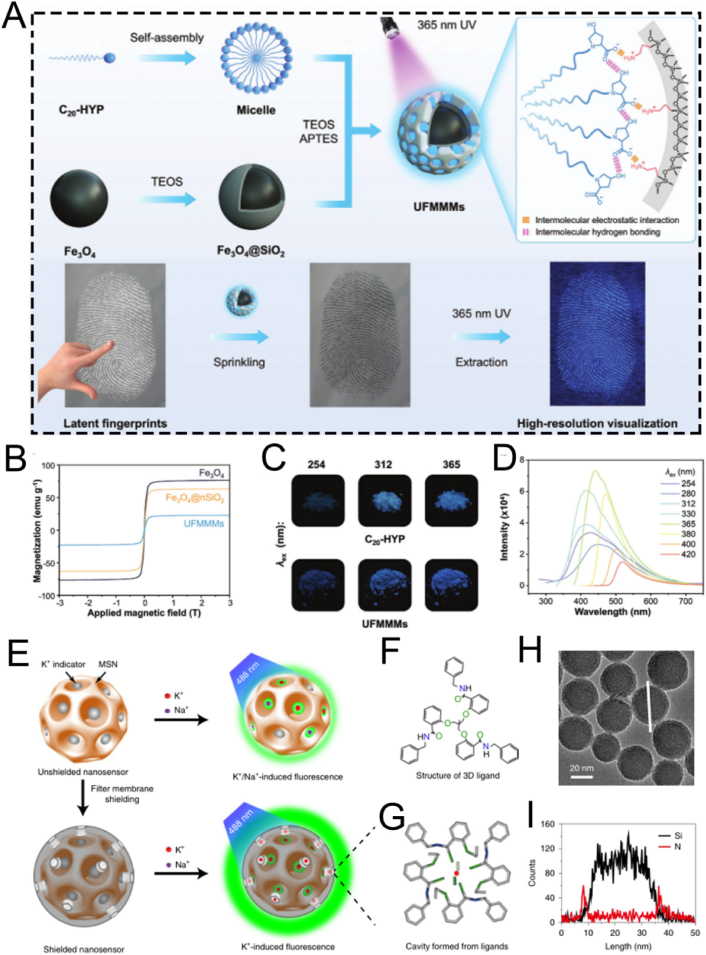
(A) Schematic illustration of UFMMM preparation and its application. (B) Magnetic hysteresis loop in Fe_3_O_4_, Fe_3_O_4_@nSiO_2_, and UFMMMs. (C) Luminescence images of UFMMM and C20-HYP powders using different UV radiation. (D) Emission spectra of UFMMM solids with different refractive indices. (E) Schematic diagram of the design of the K^+^ nanosensor. (F) Chemical structure of 3D ligands in filter membranes. (G) Chemical structure of a filter membrane with micropores filled with K^+^. (H) EDS was used to reveal the elemental distribution and confirm the schematic of the shielded nanosensor structure. (I) Representative EDS elemental analysis. (A–D) Reproduced with permission [167]. Copyright 2024, Wiley. (E–G) Reproduced with permission [168]. Copyright 2020, Springer Nature.

For example, Liu and co-workers developed innovative and cost-effective MSNs-based unconventional fluorescent magnetic mesoporous microspheres (UFMMMs). They employed designed amphiphilic non-traditional luminescent aggregate micelles (N-eicosanoyl-hydroxyproline, C20-HYP) as templates for visualizing latent fingerprints (Fig. 11A) [167]. The synthesized UFMMMs exhibited distinct blue unconventional fluorescence and had an average diameter of around 650 nm. This property enabled the clear delineation of level 1–3 characteristics of latent fingerprints on various substrates. The UFMMMs integrated subtle luminescence, paramagnetism, and excellent dispersion, demonstrating outstanding performance in fingerprint imaging (Fig. 11B–D). In the UFMMMs, intermolecular interactions between C20-HYP and amino groups enhanced the fluorescence intensity by increasing electron delocalization and the proportion of π-electrons. Specifically, in UFMMMs, the fluorescence was augmented through intermolecular interactions between C20-HYP and amino groups, which increased the proportion of π-electrons and promoted electron delocalization. This study provides a novel perspective for the controllable fabrication of fluorescent probes using non-traditional luminescent materials.

The concentration of extracellular potassium ions exerts a significant influence on the membrane potential of neurons, thereby modulating their physiological activity. Fluctuations in the levels of potassium ions have been associated with various neurological disorders, including epilepsy and Alzheimer's disease. Consequently, the selective detection of extracellular potassium ions holds great promise for enhancing disease monitoring. However, existing optical reporters encounter challenges in detecting subtle fluctuations in potassium levels, especially in freely moving animals. They are also highly susceptible to interference from sodium ions. Ling et al. developed a highly potent and specific MSN-based potassium nanosensor for monitoring dynamic potassium fluctuations in the brains of freely ranging mice during epileptic seizure activity [168]. Specifically, the optical potassium indicator was encapsulated within MSNs, which were functionalized with an ultrathin, potassium-selective membrane. This engineered membrane functioned as a selective barrier, effectively impeding the diffusion of other cations while enabling the specific capture of potassium ions (Fig. 11E–G). The uniformly thick film coating the MSNs in the nanosensor was visualized using TEM image. This was further corroborated by energy-dispersive X-ray spectroscopy (EDS)-based elemental line scanning analysis conducted on representative MSNs (Fig. 11H and I). The sustained advancement of NIR emissive K^+^ nanosensors, capitalizing on their exceptional tissue penetration capabilities, holds great promise for facilitating the precise detection of epileptic foci during whole-brain imaging. These nanosensors can be further engineered to encapsulate antiepileptic drugs, enabling the on-demand and specific release of drugs at the site of epileptic foci lesions. Collectively, this MSNs-based nanosensor has the potential to revolutionize the diagnosis and treatment of epilepsy. By providing non-invasive localization of seizure origins, it may potentially reduce the necessity for invasive surgical resection procedures.

### 4.2 Bioimaging

The advancement of MSN as a therapeutic platform has also expedited its utilization in a diverse array of diagnostic modalities, including fluorescence imaging, magnetic resonance imaging (MRI), ultrasound (US) imaging, and photothermal imaging [[169], [170], [171], [172], [173]]. This has been instrumental in the early detection of diseases and the refinement of treatment procedures, thus augmenting therapeutic effectiveness. For example, Li et al. devised a nanomedicine based on MSNs, namely CMSN@SRT@Anti, which targets macrophages for diagnostic fluorescence imaging and the regression of atherosclerotic progression [174]. Significantly, *in vitro* experiments have shown that CMSN@SRT@Anti can be more efficiently phagocytosed by macrophages in comparison to CMSN@SRT. Additionally, the fluorescence signal of inflammatory macrophages was markedly enhanced, presumably due to the upregulation of CD36 expression on their surface. CMSN@SRT@Anti exhibited a more potent and distinct effect in suppressing macrophage foam cell formation, suggesting that targeted drug delivery using functionalized MSNs can substantially enhance the therapeutic efficacy of SRT1720. *In vivo* experiments revealed that a distinct and persistent fluorescence signal was detected in atherosclerotic mice treated with CMSN@SRT@Anti. Compared to the control group, there was a significant reduction in the aortic plaque area. Collectively, the engineered nanomedicines CMSN@SRT@Anti showcase exceptional diagnostic fluorescence imaging capabilities and hold great promise for the precise identification and targeted treatment of atherosclerosis.

Notwithstanding the distinctive detection sensitivity of optical imaging techniques, their contrast within anatomical soft tissues remains relatively low, and their tissue penetration capacity is restricted. Consequently, this circumscribes the resolution of the spatial distribution of inflammatory tissues [175,176]. Such limitations give rise to significant constraints in attaining accurate quantification and detailed stratified analysis of the imaging outcomes. To address this challenge, MRI presents itself as an optimal alternative. MRI offers outstanding soft tissue contrast, excellent spatial resolution, freedom from ionizing radiation, and an unrestricted signal penetration depth [[177], [178], [179], [180]].

**Fig. 12:**
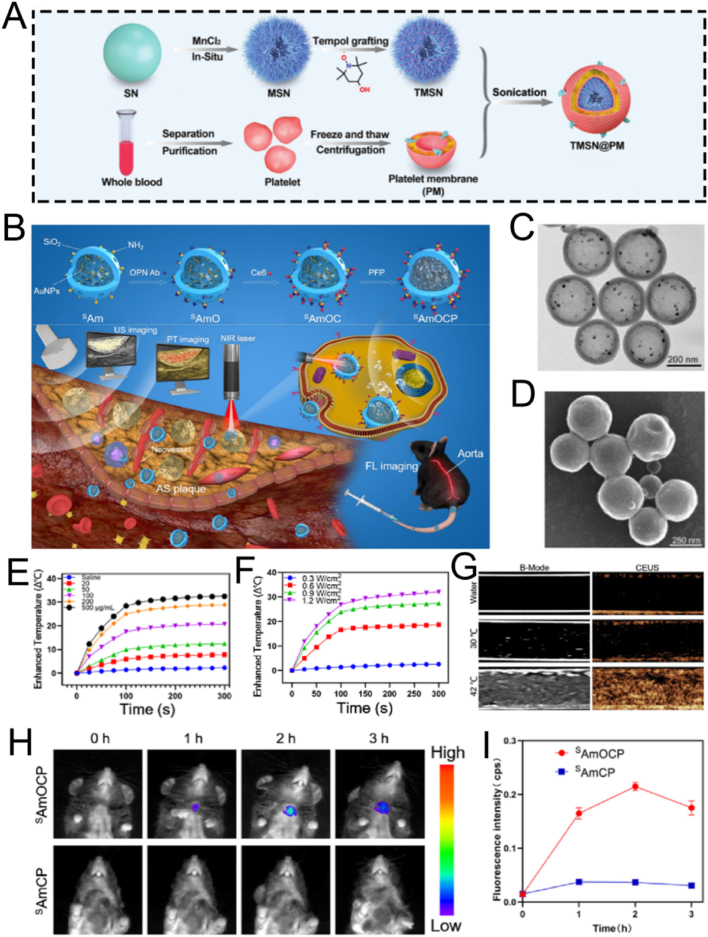
(A) Schematic diagram of the fabrication of TMSN@PM for inflammation therapy and therapeutic monitoring. (B) Schematic illustration of ^S^AmOCP composition and noninvasive multimodal visualization of atherosclerotic vulnerable plaques. (C) Representative TEM image of SAm-NH_2_. (D) Representative SEM image of SAm-NH_2_. (E) The corresponding temperature change profiles of thermal imaging of aqueous ^S^AmOCP solutions of different concentrations under 808 nm laser irradiation. (F) Photothermal heating profile of 200 μg mL^−1 S^AmOCP NPs solution against laser power density. (G) B-mode and CEUS-mode *in vitro* imaging of water and ^S^AmOCP solutions under different temperatures. (H) Fluorescence intensity at the site of targeted plaques in atherosclerotic model mice after targeted and untargeted NPs. (I) Curve of aortic arch temperature over time after near-infrared laser irradiation. (A) Reproduced with permission [181]. Copyright 2022, Wiley. (B–I) Reproduced with permission [182]. Copyright 2023, Elsevier.

Deng and colleagues developed a novel nanotherapeutic agent, TMSN@PM, which consists of a platelet membrane (PM) coating, a template, and manganese-doped MSNs. This agent is designed for real-time ratiometric MRI to alleviate inflammation (Fig. 12A) [181]. The PM endows TMSN@PM with the ability to specifically target inflammatory lesions, which can be verified by fluorescence imaging using Cy5-labeled carboxyl groups. TMSN@PM can scavenge excessive ROS, thus alleviating inflammation. Simultaneously, it enhances the efficacy of MRI through the degradation-triggered release of manganese ions. The change in relaxation rate shows an approximately linear relationship with ROS concentration, serving as a reliable biomarker for evaluating the severity of inflammation. This nanotheranostic agent, based on a non-invasive imaging strategy, has the potential to predict the therapeutic efficacy in inflammation and is expected to expedite precision medicine in terms of prognostic stratification and treatment planning.

Indeed, a single imaging modality faces challenges in identifying the key components of vulnerable atherosclerotic plaques. Additionally, it is inadequate for effectively distinguishing between stable and vulnerable plaques at the molecular level. To overcome these obstacles, the development of novel multimodal imaging strategies is essential for the accurate detection of atherosclerotic plaques. This is of great significance in preventing the formation and progression of vulnerable plaques.

For instance, Gao et al. ingeniously fabricated a versatile ^S^AmOCP nanoprobe for efficient *in vivo* targeting and multimodal imaging of atherosclerotic vulnerable plaques, encompassing US, fluorescence, and photothermal modalities. This nanoprobe utilized a hollow mesoporous silica shell to encapsulate multi-core gold self-assembled nanospheres. The surface of the nanoprobe was modified with osteopontin antibody (OPN-Ab) conjugated to chlorin e6 (Ce6), while the inner cavity was filled with perfluoropentane (PFP) (Fig. 12B) [182]. TEM and scanning electron microscopy (SEM) were employed to analyze the internal structure and surface morphology of the ^S^AmOCP nanoprobe (Fig. 12C and D). The ^S^AmOCP nanoprobe was capable of positively detecting foam cells and precisely targeting atherosclerotic vulnerable plaques within a living organism. Given the enhanced localized surface plasmon resonance effect imparted by the self-assembled particles, which endows it with excellent photothermal conversion properties, the encapsulated liquid PFP transformed into numerous microbubbles upon 808 nm laser irradiation and overheating of the atherosclerotic region (Fig. 12E and F). The elevated temperature further facilitated microbubble generation, thus significantly enhancing the US imaging ability of the nanoprobe (Fig. 12G). The ^S^AmOCP nanoprobe demonstrated remarkable fluorescence imaging capabilities *in vivo*, facilitating the precise detection of atherosclerotic plaques at the molecular level (Fig. 12H and I). In summary, the MSNs-based ^S^AmOCP nanoprobe, which integrates US, fluorescence, and photothermal multimodal imaging functions, represents a promising development for the clinical identification of atherosclerotic vulnerable plaques.

## 5 Therapeutic applications of MSN-based nanoplatforms

### 5.1 Drug delivery

Irrespective of the type of nanocarrier utilized, the pivotal aspect of nanoparticles in drug delivery lies in their ability to precisely deliver the therapeutic payload to specific target lesions within the body. This enables the optimization of therapeutic outcomes while minimizing adverse effects [[183], [184], [185], [186], [187]]. In this regard, the design of nanocarriers demands meticulous consideration of their biological interactions and behaviors throughout the drug delivery process [[188], [189], [190]]. Consequently, any engineered MSNs intended for drug delivery must factor in their biocompatibility, accurate targeting capabilities, and the reduction of side effects.

**Fig. 13:**
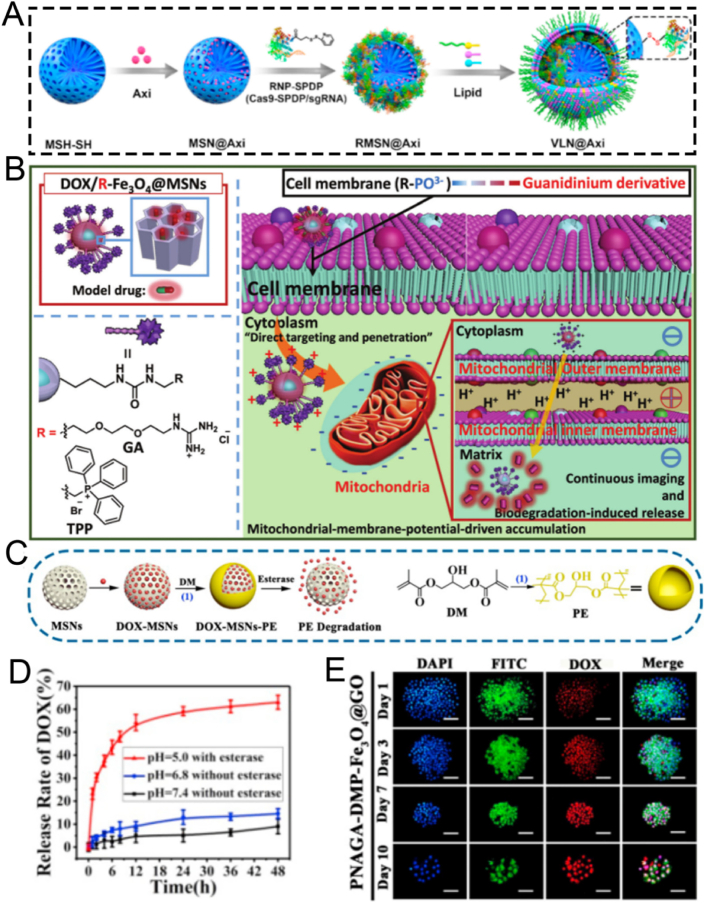
(A) Schematic diagram of the fabrication of VLN@Axi. (B) Schematic diagram of DOX/GA-Fe_3_O_4_@MSNs for mitochondria targeting. (C) Schematic diagram of the DOX-MSNs-PE preparation. (D) Cumulative release curves of DOX from DOX-MSNs-PE at different pH conditions. (E) Confocal fluorescence microscopy pictures from cells after various times through PNAGA-Fe_3_O_4_@GO hydrogel treatment. (A) Reproduced with permission [191]. Copyright 2020, Elsevier. (B) Reproduced with permission [192]. Copyright 2018, The Royal Society of Chemistry. (C–E) Reproduced with permission [193]. Copyright 2020, Elsevier.

Liu et al. developed a multifunctional Axi-loaded MSNs-based virus-like nanoparticle, designated as VLN@Axi, for the synergistic delivery of small-molecule drugs and the CRISPR/Cas9 system to achieve efficient treatment of malignant tumors (Fig. 13A) [191]. Specifically, VLN@Axi featured a core-shell architecture. The core consisted of MSNs loaded with both small-molecule drugs and the CRISPR/Cas9 system, while the outer layer was encapsulated by a lipid shell. Significantly, this structural design conferred VLN@Axi with outstanding biocompatibility. Upon reaching the tumor sites, VLN@Axi precisely released the small-molecule drugs and the CRISPR/Cas9 system, enabling the synergistic modulation of multiple cancer-related pathways. By incorporating a single guide RNA (sgRNA) targeting the programmed death ligand 1, along with axitinib, VLN@Axi effectively disrupted various immunosuppressive pathways and significantly inhibited the growth of melanoma *in vivo*. Besides this, VLN@Axi demonstrated an extraordinary capacity to co-deliver virtually any combination of small-molecule drugs and sgRNAs to tumor regions. This highlights its exceptional potential as a versatile platform for innovative combination therapies aimed at combating malignant cancers.

Mitochondrial dysfunction is intricately intertwined with the initiation and advancement of tumors, thereby highlighting the crucial significance of developing mitochondrial-targeted tumor therapeutic strategies. In this context, Jung and co-workers reported a precise nanoplatform, namely DOX/GA-Fe_3_O_4_@MSNs, which is composed of a mitochondria-targeting guanidinium derivative (GA) and the anticancer drug DOX for highly efficient cancer treatment (Fig. 13B) [192]. Significantly, the inclusion of DOX conferred fluorescence visualization capabilities upon the nanocarrier. The selective targeting properties of DOX/GA-Fe_3_O_4_@MSNs and their rapid accumulation within the mitochondria of tumor cells were successfully verified through colocalization studies using confocal laser scanning microscopy (CLSM), TEM, and fluorescence spectroscopy analysis of isolated mitochondria. DOX/GA-Fe_3_O_4_@MSNs demonstrated excellent biocompatibility with no substantial cytotoxicity, positioning them as a promising and safe drug delivery platform for targeting mitochondria in cancer therapy.

To alleviate systemic side effects and boost the efficacy of tumor treatment, integrating MSNs-based drug delivery carriers into hydrogels emerges as an outstanding therapeutic approach. For example, Chen e*t al*. fabricated a magneto-optical dual-responsive hydrogel, PNAGA-DMP-Fe_3_O_4_@GO, which consists of thermosensitive poly(N-acryloyl glycinamide) (PNAGA), Dox-loaded and polyester (PE)-coated MSNs (DOX-MSNs-PE), and Fe_3_O_4_ nanoparticles grafted onto graphene oxide (GO). This hydrogel was designed to precisely deliver drugs for specific tumor therapies (Fig. 13C) [193]. Under various magnetic field and/or NIR irradiation conditions, Fe_3_O_4_ nanoparticles and GO function as magnetothermal and photothermal agents, respectively. They induce different degrees of chain movement in PNAGA, thereby facilitating thermotherapy. This strategy not only allows for intertumoral injection by triggering a gel-sol transition in the hydrogel through preheating but also enables the regulation of the responsive release profile of DOX-MSNs-PE nanocarriers to meet the dynamic requirements of diverse patients and tumors (Fig. 13D). Additionally, the DOX-MSNs-PE nanocarriers can be internalized by surrounding tumor cells. Once inside, they release the drug upon the rapid hydrolysis of the PE coating triggered by esterase, thus enabling precise chemotherapy (Fig. 13E). *In vivo* experiments revealed that the hydrogel PNAGA-DMP-Fe_3_O_4_@GO, integrating chemotherapy and thermotherapy, effectively inhibited tumor growth and eradicated more than 90 % of tumor cells. This finding positions it as a highly promising candidate for precision anti-tumor therapy.

Compared with traditional MSNs, hollow mesoporous silica nanoparticles (HMSNs) possess an internal cavity structure that significantly enhances drug loading efficiency. Therefore, HMSNs demonstrate greater application potential in the field of drug delivery. Wang and colleagues developed a redox- and enzyme-responsive drug delivery system HMSN-SS-CD_PEI_@HA based on carbon dot-coated HMSNs for targeted drug delivery [194]. CD_PEI_ nanoparticles prepared employing polyethyleneimine (PEI) were grafted onto the pore surfaces of HMSNs *via* disulfide bonds, serving as “gatekeeper molecules" to seal the pores and efficiently encapsulate the drug within the cavity. Further grafting hyaluronic acid (HA) onto the surface of HMSNs facilitated targeted drug delivery and controlled drug release, while also enhancing the stability of the nanocarrier. Notably, the engineered HMSN-SS-CD_PEI_@HA exhibits good biocompatibility, with negligible cytotoxicity. Owing to the specific binding affinity between HA and the CD44 receptor, HMSN-SS-CD_PEI_@HA could be efficiently internalized by A549 cells with high CD44 expression through CD44 receptor-mediated endocytosis, thereby achieving targeted drug delivery. In summary, the development of a dual redox- and enzyme-responsive targeted drug delivery system based on HMSNs, along with the successful preparation of HMSN-SS-CD_PEI_@HA, offers a promising nanodelivery platform with significant potential for cancer therapy applications.

### 5.2 ROS scavenging

ROS are regarded as reactive biological molecules encompassing H_2_O_2_, •OH, and O_2_^•−^. These species play crucial roles in regulating diverse cellular metabolic processes, cellular progression, and pathophysiological events within biological systems [[195], [196], [197]]. However, an overabundance of ROS can be detrimental to cells. Specifically, they can potentially damage cell membranes, mitochondrial membranes, and DNA, thereby initiating a series of inflammatory diseases. Consequently, the scavenging of ROS emerges as an effective therapeutic approach for numerous inflammatory disorders, including inflammatory bowel disease, rheumatoid arthritis, and myocardial infarction.

**Fig. 14:**
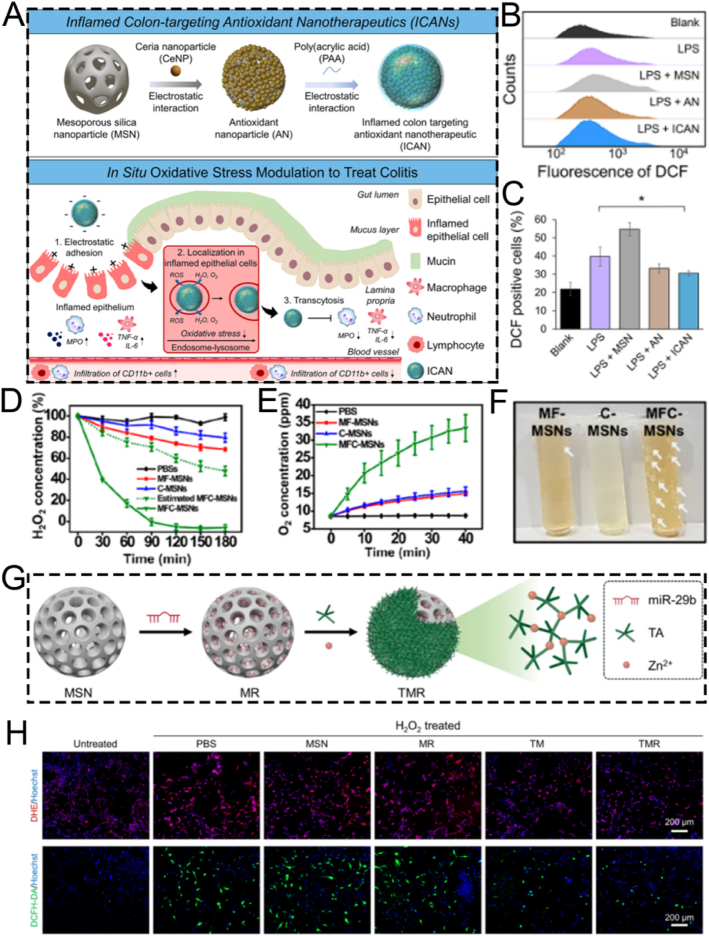
(A) Schematic diagram of the synthesis and application of inflammatory colon targeted antioxidant nanotherapeutics (ICANs). (B) Flow cytometry analysis of intracellular ROS clearance assay by DCF-DA in LPS-treated RAW 264.7 cells and (C) the corresponding quantitative analysis. (D) H_2_O_2_ degradation profiles at pH 7.4 in the existence of MF-MSN, C-MSN and MFC-MSN. (E) O_2_ production profile in 1 M H_2_O_2_ solution under physiological circumstances in the presence of NP. (F) MFC-MSNs produced significantly more O_2_ bubbles than MF-MSNs and C-MSNs after 1 h of incubation in 1 M H_2_O_2_. (G) Schematic diagram of the fabrication of TMR NPs. (H) Typical fluorescence images of cells co-cultured to diverse NPs in medium comprising 100 μmol/L H_2_O_2_. (A–C) Reproduced with permission [198]. Copyright 2023, American Chemical Society. (D–F) Reproduced with permission [199]. Copyright 2019, American Chemical Society. (G–H) Reproduced with permission [200]. Copyright 2025, Elsevier.

For instance, Kim et al. put forward the colon-targeted antioxidant nanotherapeutics ICANs, which are composed of MSNs, poly(acrylic acid), and ROS-scavenging ceria nanoparticles. These ICANs are designed to treat inflammatory bowel disease by regulating oxidative stress levels (Fig. 14A) [198]. By incorporating cerium nanoparticles, ICANs display high ROS-scavenging capabilities. Even after being incubated in simulated gastrointestinal fluids, they can maintain their efficacy (Fig. 14B and C). In a dextran sulfate sodium-induced mouse model of inflammatory bowel disease, orally administered ICANs show enhanced adhesion to the inflamed colon tissue. This targeted approach improves the intestinal microenvironment by modulating redox homeostasis and reducing inflammatory cell infiltration, thereby suppressing colitis-related immune responses. Overall, the non-invasive ceria-loaded ICANs hold the potential to treat inflammatory bowel disease by scavenging ROS and regulating oxidative stress levels in colitis.

Given the fact that cerium dioxide nanoparticles have the ability to promote macrophage M2 polarization by scavenging ROS in inflammatory diseases, Hyeon and colleagues designed a nanostructure, namely MFC-MSNs. This nanostructure consists of manganese ferrite and cerium dioxide nanoparticles anchored on MSNs. The design aims to synergistically inhibit ROS and generate O_2_, thereby facilitating the reprogramming of M1-M2 macrophages for the treatment of rheumatoid arthritis [199]. MFC-MSNs exhibited a synergistic effect in O_2_ generation and ROS scavenging. This can be attributed to the superoxide dismutase and catalase (CAT)-like activities of cerium nanoparticles (Fig. 14D–F). This effect effectively inhibits the generation of the intermediate •OH during the Fenton reaction mediated by manganese ferrite NPs for O_2_ production, thus promoting the reprogramming of M1-M2 macrophages. Intra-articular injection of MFC-MSNs was found to alleviate hypoxia, inflammation, and joint pathological changes in a rat model of rheumatoid arthritis. MSNs can function as a drug delivery vehicle for the controlled release of the antirheumatic drug methotrexate, further enhancing the therapeutic efficacy of MFC-MSNs. This study highlights the significant therapeutic potential of MFC-MSNs. By scavenging ROS and generating O_2_, MFC-MSNs can effectively facilitate macrophage reprogramming, offering a promising approach for the treatment of rheumatoid arthritis.

Excessive ROS-induced oxidative stress is not merely a pathological hallmark of inflammatory bowel disease and rheumatoid arthritis. Indeed, sustained oxidative stress can be accountable for myocardial fibrosis and can even trigger myocardial infarction. Consequently, ROS scavenging emerges as a viable strategy for alleviating myocardial infarction. Zhang et al. engineered a multifunctional MSNs-based injectable hydrogel-hinged nanocomposite (TMR) incorporating microRNA-29b (miR-29b) mimics with antifibrotic properties, antioxidant tannic acid (TA), and zinc ions (Zn). This nanocomposite is designed to scavenge ROS for the efficient treatment of myocardial infarction (Fig. 14G) [200]. Hydrogels facilitate the prolonged retention of nanocomposites within the infarcted myocardial region and enable the gradual release of nanoparticles during degradation. Significantly, the outer TA/Zn complex is capable of scavenging ROS, thereby inhibiting cell apoptosis (Fig. 14H). Subsequently, the dissociation of the TA/Zn complex results in the release of the encapsulated miR-29b mimic, which can suppress the activation of cardiac fibroblasts and the production of collagen, thus alleviating the progression of fibrosis. Additionally, the released miR-29b mimic reduces the recruitment of cardiac fibroblasts and collagen generation, further mitigating the progression of fibrosis.

In summary, this MSNs-based nanocomposite, endowed with the ability to scavenge ROS, effectively reduced the infarct volume and potentially enhanced cardiac function. These findings underscore its potential as a synergistic therapeutic approach for the recovery of infarcted myocardium.

### 5.3 Immune evasion

Building upon the biomimetic principles established in Section 3.2, immune evasion therapy shows great potential by harnessing the functional modification of biomimetic nanoparticles to substantially improve biocompatibility and optimize biointerface performance [[201], [202], [203]]. Specifically, the strategies discussed in this section aim to prolong the *in vivo* circulation of nanoparticles, enabling enhanced delivery of therapeutic agents to target sites by avoiding premature clearance by the immune system. Biocompatibility is typically utilized to comprehensively assess the biological behavior of nanoparticles and explore their potential to trigger adverse reactions in the body [204,205]. In fact, silica-based materials demonstrate favorable compatibility. Nevertheless, to optimize their *in vivo* applications, it is essential to further enhance their biocompatibility through functionalization modifications for more effective immune evasion therapy [206,207].

**Fig. 15:**
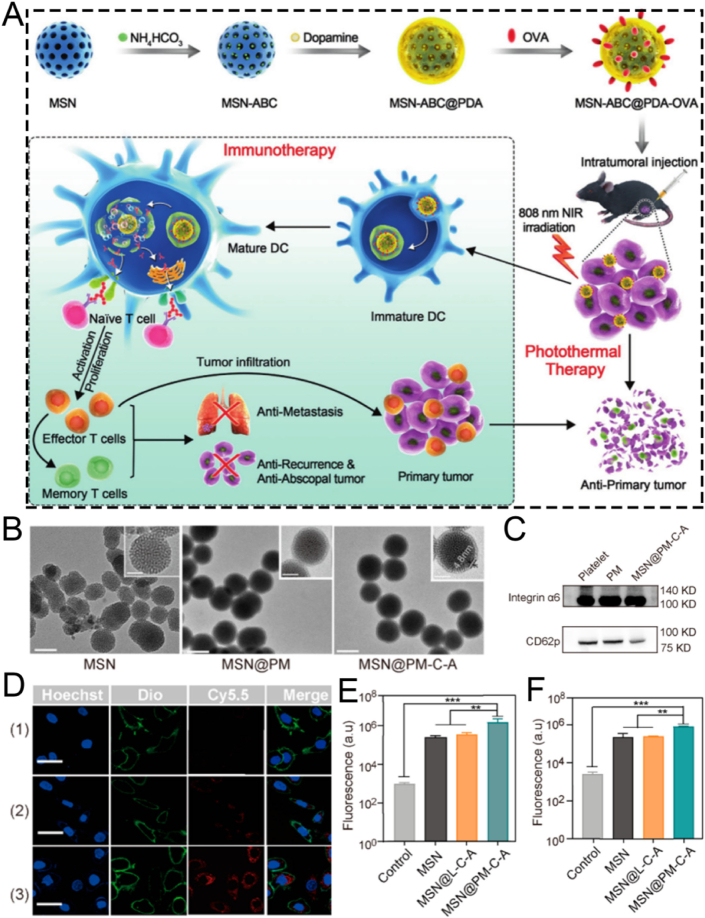
(A) Schematic diagram of the fabrication and application of nanovaccine MSNs-ABC@PDA-OVA. (B) Representative TEM images of various NPs. (C) Western blot analysis of platelet-associated membrane proteins in different substances. (D) Confocal images of V-HUVEC treated for 1 h with different nanomaterials. (E) Flow cytometry analysis of different nanomaterials after 0.5 h incubation with VHUVEC. (F) Flow cytometry analysis of different nanomaterials after 1 h incubation with VHUVEC. (A) Reproduced with permission [208]. Copyright 2021, Wiley. (B–F) Reproduced with permission [215]. Copyright 2021, American Chemical Society.

For instance, Zhang et al. developed a photothermal agent, a polydopamine (PDA)-modified biocompatible MSNs-based nanoplatform designated as MSNs-ABC@PDA-OVA. This nanoplatform incorporated the model antigen ovalbumin (OVA) and ammonium bicarbonate, an antigen release promoter, for efficient photothermal-immunotherapy against melanoma (Fig. 15A) [208]. The fabricated MSNs-ABC@PDA-OVA nanoplatform exhibited outstanding photothermal properties, enabling the effective eradication of primary tumors. Upon laser irradiation, the MSNs-ABC@PDA-OVA nanoplatform expedited the rapid release of antigens and their escape from endosomes, thereby enhancing the activation and maturation of dendritic cells. This enhancement promoted their migration to tumor-draining lymph nodes and initiated a potent anti-tumor immune response. A single administration of MSNs-ABC@PDA-OVA in combination with one session of PTT successfully eradicated melanoma, achieving a cure rate of up to 75 %. This treatment also induced a robust immune memory, effectively inhibiting tumor recurrence and lung metastasis. In conclusion, this study presents a highly efficient and promising synergistic PTT-immunotherapy strategy that can be employed for the treatment of melanoma.

PDA exhibits exceptional photothermal conversion efficiency, effectively transforming near-infrared (NIR) light into heat for localized hyperthermia, enabling it an ideal material for PTT. Moreover, the photothermal properties of PDA can facilitate catalytic reactions, thereby further enhancing its anti-tumor efficacy. In light of this, Zhao's team constructed a biocompatible PDA-coated nanoplatform GOx@Fe-DMSN@PDA (GFDP) incorporating Fe^2+^ ions into MSNs and loading glucose oxidase (GOx) for enabling multifunctional synergistic therapy [209]. The incorporation of Fe^2+^ ions significantly improved the biodegradability of GFDP, mitigating immunogenicity resulting from prolonged retention. Simultaneously, GOx depleted intracellular glucose and generated H_2_O_2_ within tumor cells, thereby inducing tumor “starvation therapy” and facilitating cascade catalytic reactions. Additionally, the PDA coating endowed nanoparticles with dual immune evasion capabilities: on one hand, it effectively reduced phagocytosis by macrophages; on the other hand, the excellent adhesive properties of PDA enhance the dispersion stability of nanoparticles in blood circulation. In summary, GFDP represents a highly promising multimodal nanoplatform for combination therapy, seamlessly integrating multiple treatment strategies-including starvation therapy, PTT, and cascade catalysis-for enhanced therapeutic efficacy.

Recent investigations have demonstrated that platelet membrane-coated nanoparticles, in which the PDA has been modified to exhibit excellent biological properties, can effectively prevent rapid clearance from the bloodstream and avoid eliciting an immune response [210]. Platelet membrane-coated nanoparticles are characterized by exceptional biocompatibility and homologous targeting abilities, allowing them to actively target tumors [[211], [212], [213], [214]]. This is mainly ascribed to the specific recognition of receptors on the surface of tumor vascular endothelial cells by the membrane molecules of these nanoparticles.

In light of this, Nie and his colleagues designed a platelet membrane-coated MSNs-based nanocomposite for the co-delivery of the vascular disrupting agent, combretastatin A4 (CA4), and the anti-angiogenic agent apatinib (Apa) to enhance tumor vascular damage (Fig. 15B and C) [215]. MSNs are highly efficient and protective in encapsulating hydrophobic anticancer drugs, allowing for controlled release and on-demand delivery [[216], [217], [218]]. CA4 specifically disrupts the vascular system of tumor tissues by binding to tubulin, while Apa is a widely used small-molecule drug that potently inhibits the tyrosine kinase activity of vascular endothelial growth factor receptor-2 [219,220]. The platelet membrane not only endows the nanocomposite with tumor-targeting properties and excellent biocompatibility but also promotes its self-enrichment in tumor tissues through specific adhesion to disrupted blood vessels *via* membrane surface receptors (Fig. 15D–F). Consequently, the targeted tumor attack and intertumoral vascular destruction synergistically complement and reinforce each other, enabling the MSN@PM-C-A nanocomposite to exhibit remarkable antitumor efficacy.

### 5.4 Gene therapy

Gene therapy, which directly targets the molecular underpinnings of diseases, exhibits transformative potential and shows great promise for the treatment of genetic disorders [221,222]. Nevertheless, the primary challenges encountered in gene therapy revolve around the optimal delivery of therapeutic genetic agents into target cells, surmounting biological barriers, and regulating toxicity and immune responsiveness [[223], [224], [225]]. MSNs are considered a potential solution to these challenges due to their unique structural characteristics [[226], [227], [228]]. In recent years, MSNs have attracted extensive attention as a promising nanocarrier for the efficient delivery of nucleic acids [229,230].

**Fig. 16:**
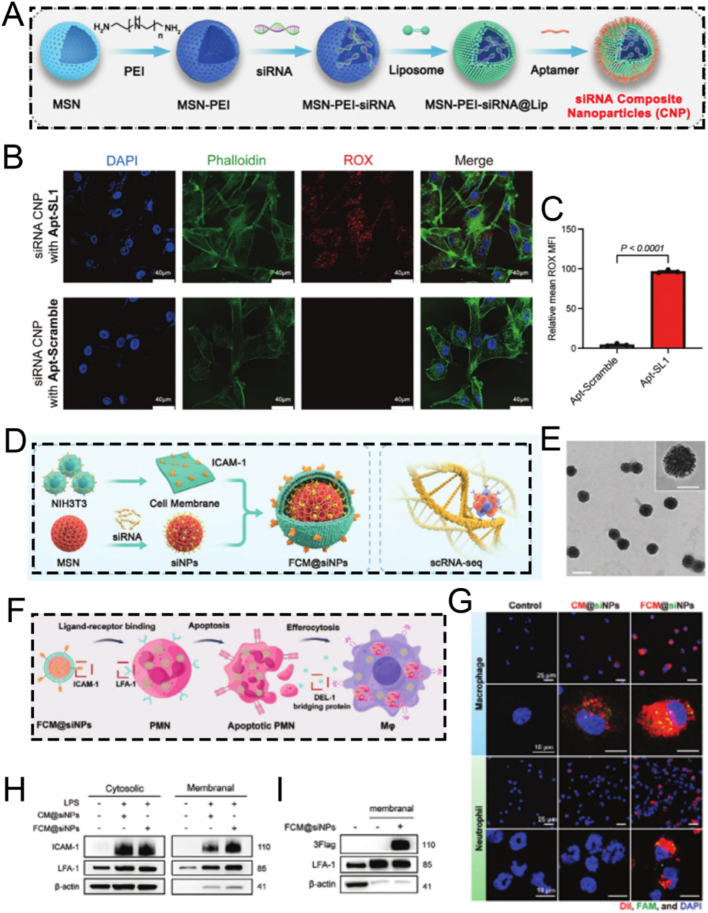
(A) Schematic illustration of preparation of siRNA CNP. (B) Confocal fluorescent images to visualize the siRNA CNP uptake through U87 MG cells and (C) corresponding quantitative data. (D) Schematic diagram of FCM@siNP delivered to mononuclear phagocytes through neutrophil airlifter. (E) Representative TEM images of FCM@siNP. (F) Schematic mechanism diagram of Neutrophil airfreighter. (G) Targeting of FCM@siNPs on Neutrophils and Macrophages using CLSM. (H) Western blot analysis of neutrophil cytoplasmic and membrane proteins. (I) Western blot analysis of the expression of PDM membrane proteins. (A–C) Reproduced with permission [231]. Copyright 2024, Wiley. (D–I) Reproduced with permission [233]. Copyright 2025, Wiley.

For example, Zhu et al. synthesized a novel MSNs-based composite nanoparticle, namely siRNA-CNP, which was modified with a lipid bilayer and the nucleic acid aptamer SL1 (Apt-SL1). This composite nanoparticle was designed to efficiently deliver small interfering RNA (siRNA) for glioblastoma gene therapy (Fig. 16A) [231]. Encapsulating siRNA within MSNs protected it from degradation and facilitated its cellular uptake. The introduction of a lipid bilayer envelope further enhanced the biocompatibility and stability of the nanoparticles. Surface modification with Apt-SL1 significantly improved the nanoparticles’ specific targeting ability towards glioblastoma, thereby enhancing the therapeutic efficacy (Fig. 16B and C). The results showed that siRNA-CNP effectively silenced the expression of CFIm25 driven by the distal poly(A) promoter in tumor cells and triggered gene reprogramming. This led to a significant attenuation of tumor growth and an increase in tumor cell apoptosis in the nude mouse model. The prepared siRNA-CNP exhibited substantial activities in tumor-suppressive gene reprogramming and glioblastoma treatment, presenting a novel and promising strategy for glioblastoma gene therapy.

For the systemic delivery of siRNA in solid tumors, the delivery vehicle is required to meet the following criteria: maintain stability in the circulatory system to prevent premature degradation, extravasate efficiently and penetrate the tumor tissue, enter target cells precisely, transport siRNA to the intracellular site of action, and finally release siRNA to effect gene silencing. Nevertheless, existing siRNA delivery vehicles, which are typically positively charged, encounter difficulties in simultaneously fulfilling all these requirements. Consequently, the efficient systemic delivery of siRNA into tumors remains a significant challenge.

To surmount the limitations of existing methodologies, Yeo and colleagues developed a MSNs-based non-cationic soft polyphenol-derived nanocapsule, namely Nanosac, to facilitate the systemic delivery of siRNA for the effective treatment of solid tumors [232]. Specifically, the MSNs were successively coated with siRNA and polydopamine. Subsequently, the sacrificial MSNs core was ablated, and finally, the siRNA-loaded Nanosac was assembled. Nanosac was capable of recruiting serum albumin and entering tumor cells *via* caveola-mediated endocytosis, thereby efficiently silencing target genes. Compared with the control groups, the soft nature of Nanosac enhanced extravasation and penetration into the tumor. As an siRNA vector targeting PD-L1, Nanosac significantly inhibited the growth of CT26 tumors through immune checkpoint blockade. These findings indicate that MSNs-based Nanosacs have the potential to be utilized as a systemic delivery platform for siRNA, enabling efficient gene therapy in solid tumors. Mononuclear phagocytes (MPs) function as vigilant sentinels. They release mitochondrial DNA (mtDNA) within the periodontal inflammatory microenvironment and engage in a complex, mainly MPs-mediated interaction with neutrophils. Given the excessive neutrophil infiltration in the inflammatory microenvironment, Song et al. designed a highly efficient and specific nanocomposite (FCM@siNPs). This nanocomposite can precisely deliver siRNA into MPs through phagocytosis to inhibit mtDNA-induced inflammation (Fig. 16D and E) [233]. The outer layer of FCM@siNPs was biomimetically functionalized with the membrane of NIH3T3 cells overexpressing ICAM-1 ligands. This nanocarrier can target neutrophils and MPs *via* the ICAM-1/LFA-1 ligand-receptor interaction (Fig. 16F–I). Considering the neutrophil transport system, the delivery efficiency of FCM@siNPs was enhanced by 120 % *in vivo* and 360 % *in vitro*. FCM@siNPs significantly disrupt the inflammatory response triggered by mitochondrial DNA release by inhibiting the expression of STING and its downstream signaling pathway in MPs. This shows promising therapeutic effects in a mouse model of inflammation. The novel neutrophil airlift approach established in this study enables efficient delivery during inflammatory surveillance. It has the potential to rapidly respond to the immune microenvironment and prevent and control excessive inflammatory reactions.

### 5.5 Dynamic therapy

#### 5.5.1 Photodynamic therapy (PDT)

PDT is a mainstream phototherapy technique with a development history spanning over a hundred years [[234], [235], [236]]. Typically, PDT comprises three components: a photosensitizer, a light source, and oxygen within the tissue [237]. Photosensitizers transfer energy to the surrounding oxygen or water in the excited state, generating ROS. This process enhances the local redox level and induces oxidative damage to cancer cells, thereby eliciting therapeutic effects. Currently, the majority of photosensitizers used in PDT are organic molecules, such as porphyrin derivatives [238]. Nevertheless, these molecules often exhibit insufficient biological stability and limited tumor selectivity, which not only impairs their therapeutic efficacy but also gives rise to severe side effects. To address these limitations, miscellaneous nanocarriers like liposomes and polymers have been explored. However, MSNs exhibit distinct advantages compared to conventional organic carriers [239]. Their rigid, porous silica framework provides superior protection for the photosensitizer payload, preventing premature degradation and aggregation. More importantly, the highly tunable surface and porous interior of MSNs allow for the co-loading of other functional agents, such as oxygen-generating catalysts (*e.g.*, MnFe_2_O_4_) or chemodynamic agents, to directly combat the core challenge of tumor hypoxia—a capability not easily achieved with simpler carriers like liposomes. Therefore, MSNs are uniquely positioned not just as delivery vehicles, but as multifunctional platforms designed to overcome the fundamental limitations of PDT.

**Fig. 17:**
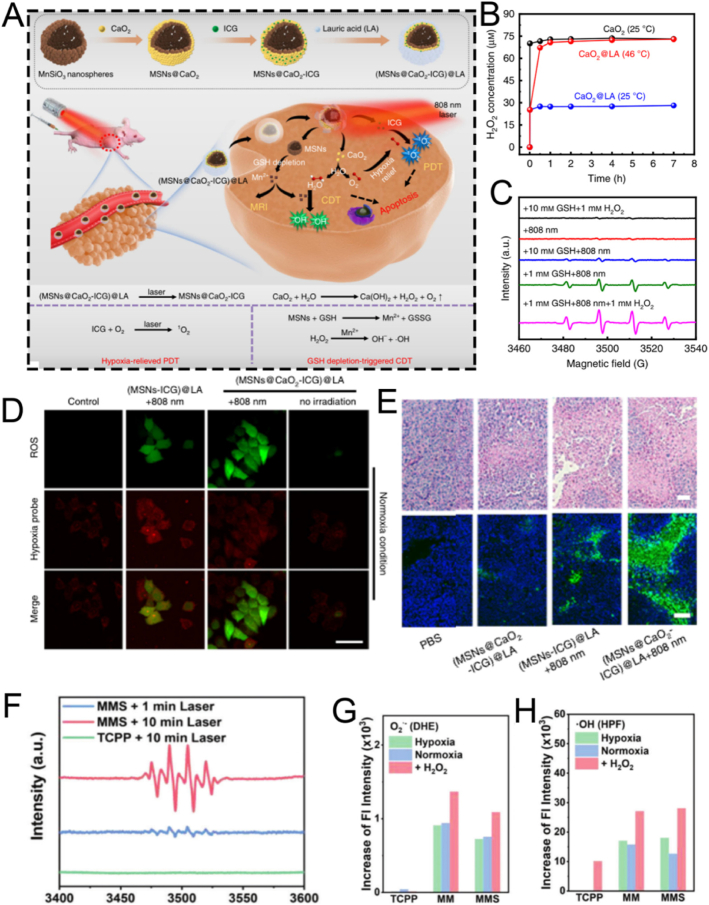
(A) Schematic illustration of preparation technique and therapeutic mechanism of (MSNs@CaO_2_-ICG)@LA NPs. (B) Cumulative H_2_O_2_ release curve. (C) Electron paramagnetic resonance (EPR) analysis of •OH generation in various treatment groups. (D) Fluorescence images exhibiting ROS and hypoxia in differently treated MCF-7 cells under normoxic conditions. (E) Tumor sections stained with H&E or TUNEL collected from various groups. (F) •OH, and O_2_^•−^ produced through TCPP and MMS NPs upon laser irradiation. (G) O_2_^•−^ production, and (H) •OH generation under various conditions. (A–E) Reproduced with permission [240]. Copyright 2020, Springer Nature. (F–H) Reproduced with permission [242]. Copyright 2023, Wiley.

For example, Dong et al. reported a self-supplying H_2_O_2_/O_2_ nanotherapeutic agent, (MSNs@CaO_2_-ICG)@LA, which consists of MSNs, CaO_2_, indocyanine green (ICG), and the phase-change material lauric acid (LA). This agent was designed to enhance the generation and reduce the expenditure of ROS production, following a strategy for synergistic photodynamic/chemodynamic therapy (Fig. 17A) [240]. Upon laser exposure, ICG generated singlet oxygen and released heat, which melted LA (Fig. 17B). The exposed CaO_2_ reacted with water to produce H_2_O_2_ and O_2_. This provided H_2_O_2_ for CDT and alleviated hypoxia for ICG-mediated PDT, thus achieving enhanced ROS generation through an open-source approach (Fig. 17C). Additionally, the depletion of GSH induced by MSNs served as a “reduced consumption” mechanism by decreasing the scavenging of ROS (Fig. 17D and E). This “increasing production” and “reducing consumption” strategy effectively inhibited tumor growth. It significantly broadened the efficiency of ROS production in ROS-directed cancer PDT at multiple scales through a MSNs-based nanoagent.

The therapeutic efficacy of PDT is significantly limited by the hypoxic conditions commonly present in tumor microenvironments. This is because the mechanism of PDT critically relies on the availability of oxygen. Hyeon and colleagues designed biocompatible manganese-ferric oxide (MnFe_2_O_4_) nanoparticles attached to mesoporous silica nanoparticles (MFMSNs) to relieve tumor hypoxia and enhance the therapeutic effectiveness of PDT [241]. MFMSNs utilize MnFe_2_O_4_ nanoparticles to continuously release oxygen through the Fenton reaction. This effectively alleviates tumor hypoxia with a minimal quantity of nanoparticles and significantly improves the *in vivo* therapeutic efficacy of PDT against tumors. MFMSNs display a *T*_2_ MRI contrast effect, enabling *in vivo* tracking. These findings indicate that MFMSNs have great potential as theranostic agents in cancer therapy, integrating both therapeutic and diagnostic functions.

PDT is also widely employed to treat bacterial infections. However, the biofilm matrix and hypoxic microenvironment significantly hinder its effectiveness against bacterial biofilm infections. Therefore, in a study, Dong et al. developed a phototherapeutic nanoplatform, namely MSN@MOF/SNP nanoparticles (MMS NPs). This nanoplatform consists of the nitric oxide (NO) donor sodium nitroprusside (SNP), amino-functionalized MSN, and the porphyrin-based metal-organic framework (Ti-TCPP MOF). It enables photodynamic characteristics and NO release behavior, thereby enhancing the penetration and removal of hypoxic bacterial biofilms [242]. Within the hypoxic environment of biofilms, MMS NPs release NO to increase biofilm permeability and generate •OH and O_2_^•-^ *via* photodynamic pathways (Fig. 17F–H). Subsequently, the synergistic effect of ROS and NO gases effectively eradicates biofilm-associated infections. Indeed, NO also contributes to collagen deposition and angiogenesis *in vivo*. In conclusion, MMS NPs represent a powerful PDT therapeutic strategy for biofilm-associated infections, offering a promising alternative in the battle against biofilm-related infectious diseases.

#### 5.5.2 Sonodynamic therapy (SDT)

Building on the principle of using external energy to generate cytotoxic ROS, SDT is an additional cancer treatment approach that inflicts oxidative stress damage on cancer cells by elevating ROS levels. While PDT relies on light, SDT employs ultrasound (US) to activate specific sonosensitizers, providing the significant advantage of enhanced tissue penetration depth. These sonosensitizers then catalyze the generation of ROS within cancer cells, leading to targeted tumor ablation [243]. SDT exhibits remarkable antibacterial properties through the production of ROS, holding promising potential for application in the antimicrobial treatment of periodontitis. Nevertheless, the insufficient efficiency of ROS production remains one of the major challenges for SDT.

**Fig. 18:**
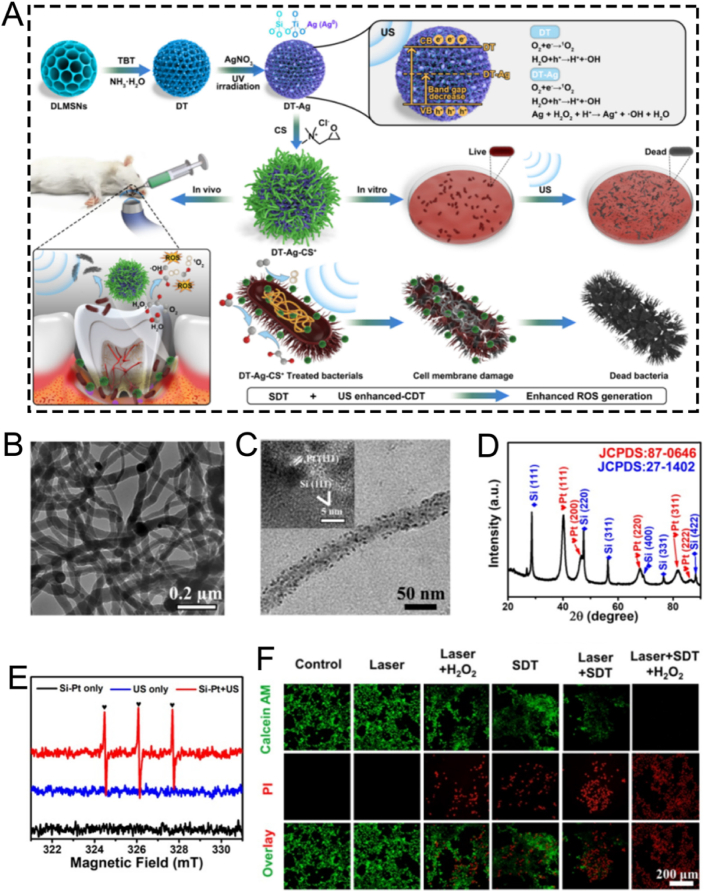
(A) Schematic diagram of the synthesis and the anti-periodontitis effect of DT-Ag-CS^+^ nanoparticles. (B) Representative TEM image of SiNWs. (C) Representative TEM image of Si-Pt nanocomposites. (D) X-ray Powder Diffractometer (XRD) of Si-Pt nanocomposites. (E) EPR spectra revealing ^1^O_2_ production through Si-Pt nanocomposites under US irradiation. (F) Confocal microscopy images of 4T1 cells stained with Calcein-AM and PI after various treatments. (A) Reproduced with permission [244]. Copyright 2023, Elsevier. (B–F) Reproduced with permission [245]. Copyright 2021, Ivyspring International Publisher.

To address this issue, Yang et al. synthesized a novel nano-sonosensitizer, DT-Ag-CS^+^, which consists of dendritic large pore mesoporous silica nanoparticles, TiO_2_, Ag, and quaternary ammonium chitosan. This was done to enhance ROS production for combating periodontitis *via* SDT/CDT synergistic therapy (Fig. 18A) [244]. They employed a porphyromonas gingivalis (P. gingivalis) and ligature-induced periodontitis rat model to assess the *in vitro* antibacterial effects of DT-Ag-CS^+^ and its *in vivo* therapeutic efficacy against periodontitis. Due to the accumulation of precious metal Ag, DT-Ag-CS ^+^ exhibited remarkable SDT and CDT therapeutic effects both *in vitro* and *in vivo*. The high positive charge of CS ^+^ on the surface of DT-Ag facilitated better penetration of DT-Ag-CS^+^ into bacterial cells. The versatile DT-Ag-CS^+^ nano-sonosensitizer shows promising clinical application prospects for the synergistic treatment of periodontitis through SDT and CDT.

US-triggered SDT offers notable advantages, including a significant penetration depth and mild phototoxicity. This enables it to address the critical limitations of phototherapy. However, the development of more efficient sonosensitizers is still necessary to enhance the treatment efficacy.

In another research, Cheng et al. prepared Si-Pt nanocomposites (Si-Pt NCs) composed of silicon nanowires (SiNWs) and Pt NPs *via* an in-situ reduction technique for photothermal-enhanced SDT (Fig. 18B–D) [245]. Si-Pt NCs are capable of generating ROS under US irradiation, thereby exerting a sonodynamic therapeutic effect (Fig. 18E). Intriguingly, Si-Pt NCs can catalyze the conversion of excess H_2_O_2_ in the tumor microenvironment into ROS, thus inducing a potent CDT effect. Compared with pure Pt NPs or SiNWs, the mesoporous structure of SiNWs endows Si-Pt NCs with enhanced SDT and CDT efficacies. The mild photothermal property of Si-Pt NCs further boosts the SDT and CDT activities, enabling a combined therapeutic modality for tumor treatment (Fig. 18F). In conclusion, Si-Pt NCs, with the ability for photothermally improved SDT/CDT synergistic therapy, hold great application value in novel tumor therapy strategies.

### 5.6 Catalytic therapy

Nanocatalytic techniques refer to the action of nanozymes that adhere to the kinetics of enzymatic reactions under physiological conditions. These nanozymes catalyze the transformation of substrates by mimicking the function or structure of natural enzymes. Currently, nanozymes mainly include oxidoreductases, such as peroxidase (POD), glucose oxidase (GOx), and antioxidation-related CAT [[246], [247], [248]]. By designing nanozymes with diverse catalytic activities, it is feasible to precisely regulate the cellular redox balance, either by promoting or scavenging ROS. This enables targeted therapy in various disease models [[249], [250], [251], [252]].

Given their abundant and tunable pore structures, MSN have emerged as highly efficient nanoreactors in enzyme-mimetic therapy [[253], [254], [255], [256], [257]]. In a study by Yang et al., a metal-free Type I photosensitizer - nitrogen-doped carbon quantum dots/mesoporous silica nanoparticles (NCDs/MSN) nanohybrid with POD-like properties was developed *via* a simple one-pot method for combined PDT and enzyme-mimetic catalysis treatment [258]. Due to the narrow band gap and excellent charge separation ability of NCDs/MSN, the excited electrons in the conduction band can effectively reduce dissolved oxygen through single electron transfer under 640 nm illumination, even in a hypoxic environment, to generate O_2_^•-^, thus inducing tumor cell apoptosis. Additionally, the O_2_^•-^ generated by light induction can be further reduced through a two-electron process to produce the more toxic •OH. NCD/MSN exhibits POD-mimicking activity and can catalyze the generation of •OH from endogenous H_2_O_2_ in the tumor microenvironment, further synergistically inducing the death of 4T1 tumor cells. Consequently, this technique offers a feasible route for the large-scale synthesis of novel metal-free POD-active Type I photosensitizers. It holds the potential for the synergistic treatment of hypoxic tumors and shows promising prospects for clinical translation.

To further boost catalytic efficiency, the field has recently seen a thriving development of nanocomposites with multi-enzyme catalytic activities. Multi-enzyme catalytic cascade reactions entail the sequential and collaborative catalysis of a series of chemical reactions by multiple enzymes and play a crucial role in biological signaling and metabolic pathways. AuNPs have drawn substantial attention because of their diverse enzyme-like activities, allowing them to concurrently mimic the functions of natural GOx and POD.

**Fig. 19:**
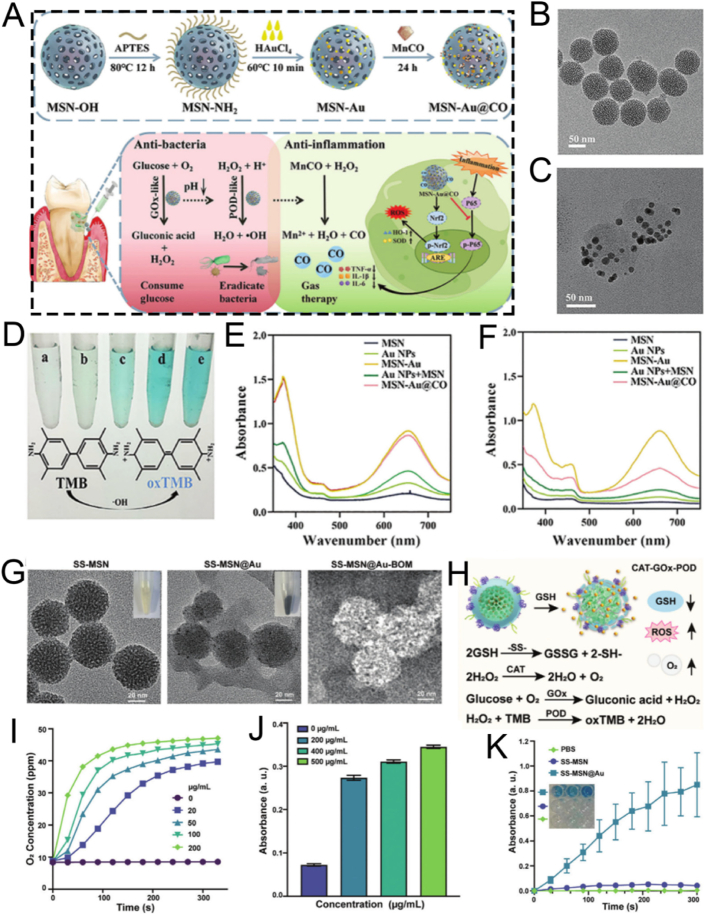
(A) Schematic diagram of the assembly of the MSN-Au@CO. (B) Representative TEM images of MSN. (C) Representative TEM images of MSN-Au@CO. (D) Schematic illustration of the TMB chromogenic reaction mechanism. (E) The GOx-like efficacy of MSN-Au@CO. (F) The POD-like efficacy of MSN-Au@CO. (G) Representative TEM images of various nanoparticles. (H) Schematic illustration of GSH-mediated degradation of SS-MSN@Au-BOM and the CAT-GOx-POD cascade catalytic system. (I) O_2_ concentration curves showing the reaction of SS-MSN@Au-BOM samples at various concentrations with H_2_O_2_. (J) Absorbance of H_2_O_2_ produced from the reaction of SS-MSN@Au with glucose at various concentrations. (K) Time-dependent absorbance of oxidized TMB (oxTMB) upon addition of H_2_O_2_ in different systems. (A–F) Reproduced with permission [259]. Copyright 2024, Wiley. (G–K) Reproduced with permission [262]. Copyright 2024, Wiley.

For instance, Deng et al. developed an efficient multi-enzyme cooperative hybrid nanoplatform (MSN-Au@CO) consisting of MS), AuNPs, and carbonyl manganese. This nanoplatform was designed to generate carbon monoxide (CO) gas for the treatment of diabetic periodontitis (Fig. 19A–C) [259]. Au NPs demonstrated GOx-like activity, enabling them to catalyze the oxidation of glucose, resulting in the production of H_2_O_2_ and gluconic acid. Additionally, AuNPs could convert H_2_O_2_ into •OH through their POD-like activity, thereby effectively eliminating bacteria (Fig. 19D–F). The CO generated in response to H_2_O_2_ synergized with AuNPs, showing a combined anti-inflammatory effect in lipopolysaccharide-stimulated macrophages. The antibacterial and anti-inflammatory properties of MSN-Au@CO were verified in a ligature-induced diabetic periodontitis rat model. This demonstrated its excellent biocompatibility and its ability to effectively reduce periodontal bone loss. In conclusion, the fabricated MSN-Au@CO utilizes a glucose-activated GOx-POD cascade catalytic effect to eradicate bacteria and combines with gas therapy to regulate the immune microenvironment, offering promising prospects for the precise treatment of diabetic periodontitis.

The natural CAT in the bacterial outer membrane (BOM) can catalyze H_2_O_2_ to generate oxygen, thereby enhancing the glucose oxidase-peroxidase (GOx-POD) catalytic cascade of Au nanozymes [260,261]. Consequently, Yue and colleagues proposed a self-oxidizing biomimetic BOM-modified nanoplatform, SS-MSN@Au-BOM, with CAT-like and GOx-POD cascade catalytic properties. This nanoplatform aims to modulate the tumor microenvironment by utilizing Au nanozymes and MSNs doped with disulfide bonds for tumor catalytic immunotherapy (Fig. 19G and H) [262]. Specifically, the Au nanozyme exhibits dual enzyme-like activities, mimicking both GOx and POD, while the BOM coating is endowed with CAT functionality (Fig. 19I–K). The SS-MSN@Au-BOM nanoplatform effectively inhibits tumor growth by catalyzing the production of cytotoxic ROS and oxygen from endogenous H_2_O_2_, simultaneously alleviating tumor hypoxia and increasing ROS levels. This catalytic action facilitates immunogenic cell death, enhances the maturation of dendritic cells, and activates T cells. This catalytic effect speeds up immunogenic cell death, the maturation of dendritic cells, and the activation of T cells. This nanoplatform exhibits excellent biocompatibility, favorable tumor-targeting ability, and a prolonged circulation time *in vivo*. As a bifunctional strategy, SS-MSN@Au-BOM can effectively disrupt the tumor microenvironment and activate a potent anti-tumor immune response. This research paves the way for subsequent clinical studies and the development of enzyme-based therapies, with the potential to reshape the landscape of cancer treatment.

### 5.7 Antibacterial application

Bacterial infections continue to pose a substantial threat to global public health. This threat is exacerbated by the increasing prevalence of antibiotic resistance. Given the complex microenvironment of bacterial infections, conventional antibiotic treatments frequently have limited effectiveness. Thus, there is an urgent need to develop novel and more effective antibacterial therapeutic approaches. MSNs, as outstanding antibiotic carriers, demonstrate unique physicochemical advantages in antibacterial therapy. Besides their delivery function, a great deal of research has focused on engineering MSNs into biologically active carriers to further enhance the synergistic effects of infection treatment.

**Fig. 20:**
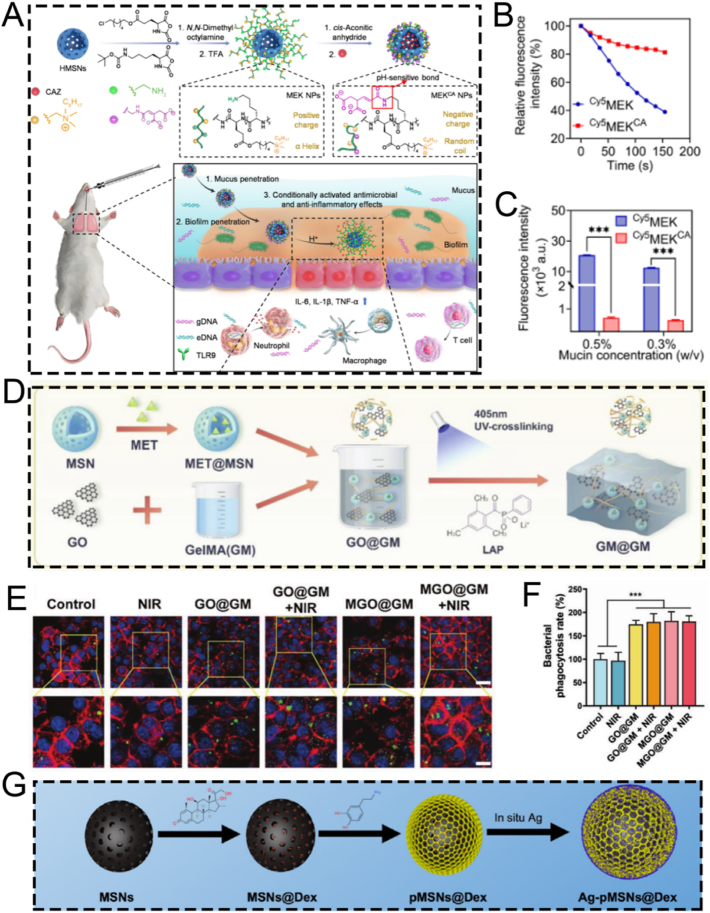
(A) Schematic diagram of IMAMs piercing mucus and biofilms for immunoantimicrobial management of chronic obstructive pulmonary disease. (B) Correlation fluorescence intensity of fluorescence images of ^Cy5^MEK and ^Cy5^MEKCA NP in CF sputum after various times of photobleaching in the circled area. (C) Fluorescent strength of ^Cy5^MEK and ^Cy5^MEKCA NP forming aggregates with mucin glycoproteins under various mucin concentrations. (D) Schematic illustration of the synthesis of MGO@GM hydrogel for the treatment of biofilm-infected diabetic wounds (BIDW). (E) Representative fluorescence confocal images of macrophages cultured with GFP Staphylococcus aureus following various treatments. (F) Detection of bacterial phagocytosis in macrophages after different treatments by smear plate method. (G) Schematic illustration of the Ag-pMSNs@Dex preparation. (A–C) Reproduced with permission [263]. Copyright 2024, American Association for the Advancement of Science. (D–F) Reproduced with permission [264]. Copyright 2023, Wiley. (G) Reproduced with permission [265]. Copyright 2023, Elsevier.

For example, Yin et al. developed immune-antibacterial molecules (IMAMs) featuring dual penetration of mucus and biofilms. These IMAMs consist of ceftazidime (CAZ) and hollow MSNs and are designed for the treatment of chronic obstructive pulmonary disease (COPD) through combined antibacterial and immunotherapy approaches (Fig. 20A) [263]. Significantly, the prepared IMAM nanoparticles were gated using charge- and conformation-switchable peptides. The peptide was encapsulated on the surface of the hollow MSNs in a negatively charged, randomly coiled conformation, sealing the pores to prevent antibiotic leakage. This also enabled the nebulized IMAMs to effectively penetrate biofilms and bronchial mucus (Fig. 20B and C). In an acidic biofilm environment, the peptide transformed into a positively charged rigid α-helical structure. This transformation enhanced its retention within the biofilm and exposed the pores for the release of the antibiotic CAZ. Additionally, the peptide was activated under specific conditions to disrupt bacterial membranes and expel bacterial DNA. It therefore acted as a synergistic agent with CAZ, enabling the efficient elimination of lung-colonising bacteria. Crucially, it inhibited the activation of Toll-like receptor 9, facilitating the subsidence of inflammation. This immune-antibacterial strategy based on MSN nanocomposites has the potential to revolutionize the current treatment paradigm for COPD.

In addition to its association with COPD, biofilm infection is also linked to the pathogenesis of diabetic wounds. Biofilm-infected diabetic wounds (BIDW) display dysregulated inflammation and abnormal activation of NLRP3 inflammatory vesicles in response to the combined effects of hyperglycemia and bacterial colonization. This leads to sustained M1-type polarization of macrophages and excessive formation of neutrophil extracellular networks (NETs). Excessive downregulation of NLRP3 through anti-inflammatory treatments may inadvertently promote the persistence of biofilm infections, thereby worsening the impaired healing of BIDW. Thus, synergistically enhancing biofilm clearance and immune regulation shows promise for achieving effective healing of BIDW. Wang et al. developed a nanocomposite hydrogel (MGO@GM) composed of gelatin methacrylate hydrogel (Gel-MA), graphene oxide (GO), and metformin-loaded MSNs to biophysically modulate NLRP3 for the treatment of BIDW (Fig. 20D) [264]. Initially, the formation of bacterial biofilms was inhibited, and mature biofilms were disrupted by GO nanosheets released from MGO@GM upon NIR irradiation (Fig. 20E and F). Simultaneously, GO activated NLRP3, inducing a pro-inflammatory response in macrophages targeting the biofilm. Subsequently, as MGO@GM continued to degrade, the released metformin reduced local hyperglycemia levels, downregulated NLRP3 activity, and inhibited NETs formation. The inflammatory microenvironment was alleviated and tissue regeneration was facilitated by the repolarization of M2-type macrophages. Overall, this MSN-based nanocomposite hydrogel offered innovative perspectives for the clinical treatment of BIDW. It achieved this by modulating NLRP3, restoring the impaired innate immune function, and synergistically promoting anti-infection and tissue repair.

Scaffolds incorporating antibacterial and osteogenic agents are considered a crucial strategy for restoring bone defects caused by osteomyelitis. Nevertheless, the simultaneous release of these two agents may lead to premature osteogenesis. Subsequently, this can result in sequestrum formation and potentially irreversible osteoporosis under pathological conditions.

To tackle this problem, in the report by Yang et al., a core/shell drug delivery nanocomposite, Ag-pMSNs@Dex, which utilized polydopamine-functionalized MSNs, was designed. This nanocomposite was loaded with the osteogenic agent dexamethasone (Dex) and antibacterial silver (Ag) nanoparticles. It was intended to regenerate bone and release drugs in a controlled manner for the effective treatment of osteomyelitis (Fig. 20G) [265]. The prepared Ag-pMSNs@Dex released Ag^+^ ions within the first 12 h, and Dex release commenced on the 5th day. Subsequently, the antibacterial properties of the nanocomposite were evaluated using Staphylococcus aureus and Escherichia coli as test strains. The Ag-pMSNs@Dex nanocomposite significantly enhanced the osteogenic differentiation of mesenchymal stem cells derived from the bone marrow of mice. In conclusion, this Ag-pMSNs@Dex nanocomposite demonstrates excellent osteogenic effects, offering a novel and straightforward approach for the efficient treatment of osteomyelitis.

### 5.8 Vaccine application

While the immune evasion strategies mentioned earlier focus on avoiding the immune system for therapeutic delivery, a related but distinct application involves using nanoparticles to intentionally engage and stimulate it. In conventional vaccine technologies, a variety of adjuvants are utilized to boost immunogenicity. This is achieved by activating dendritic cells and triggering potent antigen-specific immune responses. Adjuvants can be classified according to their immunostimulatory effects on antigen-presenting cells and their potential as delivery systems to facilitate antigen uptake. Determining the optimal combination of adjuvants and antigen carriers is one of the most challenging and crucial aspects in vaccine production. To meet these requirements, numerous nanosystems, such as polymeric or lipid nanocarriers, have been explored and evaluated. However, most of these nanosystems exhibit poor stability and are liable to degrade in the harsh gastric environment, leading to the premature release of their payload. The stability of MSNs in the intestinal transit environment makes them a promising candidate for antigen delivery systems and vaccine adjuvants [266]. As a result, studies have explored the use of MSNs for delivering various types of antigens, including antigens from gemcitabine (GEM)-resistant 4T1 cells (GEM-rAgs), surface proteins of the H1N1 influenza virus, and cytoplasmic antigens from drug-resistant Pseudomonas aeruginosa PAO1.

**Fig. 21:**
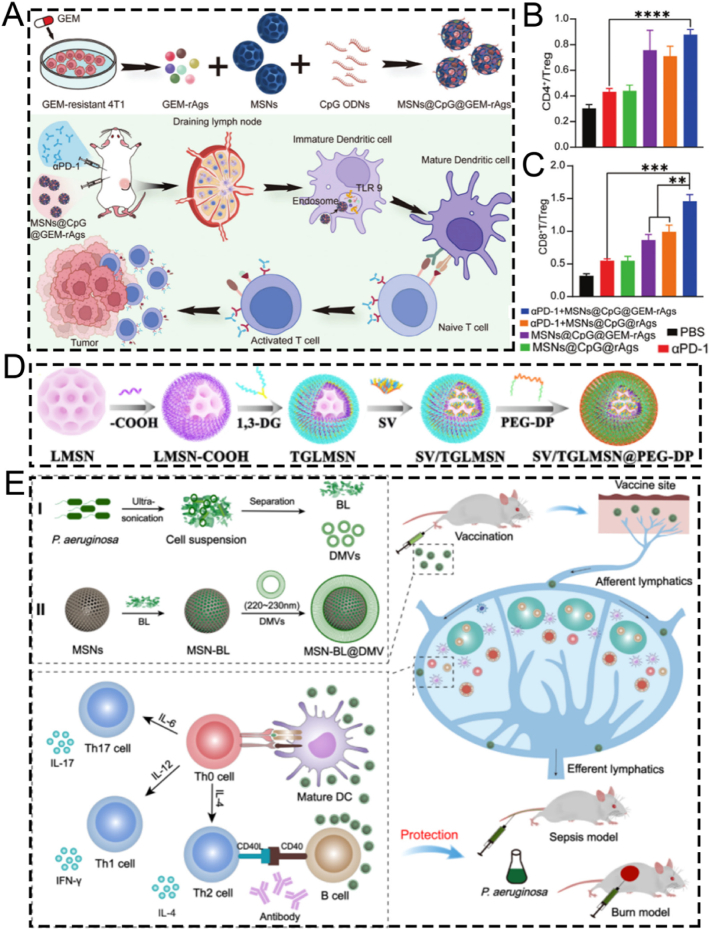
(A) Schematic diagram of the synthesis and application of the nanovaccine MSNs@CpG@GEM-rAgs. (B) Ratio of CD4^+^ T cells to T lymphocytes within the tumor. (C) Ratio of CD8^+^ T cells to T lymphocytes within the tumor. (D) Schematic illustration of the process of constructing a bionic delivery platform (SV/TGLMSN@PEG-DP) for influenza lysate vaccine. (E) Schematic diagram of a multi-antigen vaccine against Pseudomonas aeruginosa infection. (A–C) Reproduced with permission [267]. Copyright 2024, Wiley. (D) Reproduced with permission [268]. Copyright 2023, Elsevier. (E) Reproduced with permission [269]. Copyright 2022, Elsevier.

For example, Min and colleagues developed a therapeutic MSNs vaccine, namely MSNs@CpG@GEM-rAgs. This vaccine incorporates GEM-rAgs and the immunostimulant CpG oligodeoxynucleotide that targets Toll-like receptor 9. It enables the sustained release of both antigens and CpG, thereby enhancing the therapeutic effectiveness against chemotherapy-resistant tumors (Fig. 21A) [267]. The nanovaccines can efficiently target the draining lymph nodes, enhance antigen uptake by dendritic cells (DCs), and promote DC maturation. As a result, they significantly inhibit the growth of drug-resistant tumors. When combined with immunotherapy, the efficacy of the proposed nanovaccine against GEM-resistant tumors is further enhanced by increasing intra-tumor T-cell infiltration (Fig. 21B and C). The MSNs@CpG@GEM-rAgs offers a potential alternative strategy for specifically delivering GEM-rAgs to address drug resistance using cancer nanovaccines, thus broadening the prospects for clinical applications.

Oral vaccines hold great potential for preventing major infectious diseases. However, their development is hampered by diverse gastrointestinal tract barriers and a reduction in intestinal microfolded (M) cells. Inspired by the innate absorption mechanism of dietary fats, Wang et al. engineered a biomimetic MSNs-based intestinal lymphatic nanosystem, designated as TGLMSN@PEG-DP. This nanosystem features excellent intestinal epithelial permeability and lymphatic targeting ability. It is designed to deliver the H1N1 influenza split vaccine (SV), thereby effectively enhancing both mucosal and systemic immune responses to the influenza vaccine (Fig. 21D) [268]. TGLMSN demonstrates dual functionality as both an adjuvant and an antigen carrier, significantly augmenting the stimulatory effect of SV on dendritic cell maturation. The findings confirm that, compared with the administration of single oral SV, TGLMSN@PEG-DP substantially enhances SV-induced systemic and mucosal antigen-specific immune responses. This study developed tailored mesoporous silica nanocarriers for SV loading. These nanocarriers primarily exploit the intestinal epithelial cell pathway rather than relying solely on M-cell-mediated delivery to mesenteric lymph nodes. The TGLMSN@PEG-DP not only shows promise for optimizing current influenza vaccination strategies but also offers novel perspectives for the design of vaccines against mucosally transmitted pathogens.

In an additional study, Sun et al. reported an antigen-loaded, MSNs based nanovaccine designated as MSN-BL@DMV. This nanovaccine was designed to deliver the cytoplasmic antigen BL derived from the drug-resistant Pseudomonas aeruginosa PAO1 strain, aiming to effectively defend against P. aeruginosa infections (Fig. 21E) [269]. The engineered MSN-BL@DMV presented a well-defined core-shell structure. This structural feature significantly enhanced the endocytosis and maturation of DCs without the need for additional adjuvants. The MSN-BL@DMV nanovaccine, formulated using PAO1 antigens, exhibited efficient migration to the lymph nodes after subcutaneous administration. It elicited a robust and antigen-specific humoral and cellular immune response. This response conferred substantial protection against infections caused by both the PAO1 strain and another drug-resistant P. aeruginosa strain. Overall, the MSNs-based integrated antigen delivery system effectively protected mice from infections caused by various P. aeruginosa strains. This achievement paves the way for novel therapeutic strategies to combat other refractory pathogen infections.

### 5.9 Tissue engineering application

Shifting the paradigm from employing MSNs as mobile agents to treat disease, this section explores their emerging role as functional components within static scaffolds for tissue regeneration. Beyond their established roles in targeted drug delivery and diagnostics, MSNs are increasingly being leveraged as a transformative component in the field of tissue engineering. The central goal of tissue engineering is to create functional constructs that can restore or replace damaged tissues, a process that relies heavily on biomaterial scaffolds. By serving as nanoscale reservoirs for growth factors, ions, and other signaling molecules, MSNs can be integrated into scaffold matrices to create advanced composite systems. This strategy transforms the bulk scaffold into a sophisticated local delivery platform. The high loading capacity and tunable release kinetics of MSNs enable the sustained and controlled presentation of therapeutic agents, effectively mimicking the dynamic signaling environment of the native extracellular matrix. The amenability of MSNs to surface functionalization allows for targeted interactions with specific cell types or ECM components, enhancing cell adhesion and guiding differentiation pathways. This synergy between the structural scaffold and the functional nanoparticles provides an unparalleled level of control over the regenerative niche.

**Fig. 22:**
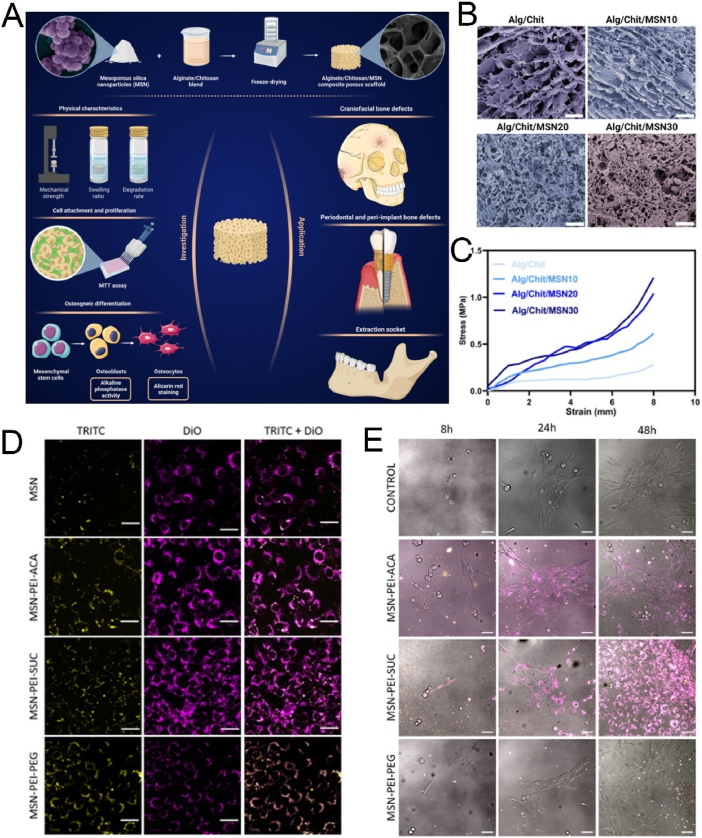
(A) Schematic diagram of Fabrication, Characterization, and Application of an Alginate/Chitosan/MSN Composite Scaffold for Bone Tissue Engineering. (B) Scanning electron microscope image of the fabricated scaffold. (C) The stress-strain curve of the freeze-dried scaffold. (D) Representative confocal microscope images of the co-incubation of MSN and DiO in 2D cultures. (E) Confocal 2.5D time-lapse images of myoblasts cultured on T-MSN/DiO-incorporated hydrogel, demonstrating nanoparticle uptake from the hydrogel and intracellular release. (A–C) Reproduced with permission [270]. Copyright 2023, Springer Nature. (D–E) Reproduced with permission [271]. Copyright 2023, Elsevier.

Addressing the clinical challenge posed by oral and craniofacial bone defects, one study fabricated a multifunctional MSNs-based scaffold integrating a matrix of alginate and chitosan (Alg/Chit) *via* the freeze-drying method for bone tissue engineering (Fig. 22A and B) [270]. The porous composite scaffolds were prepared via freeze-drying, with MSNs incorporated at varying concentrations (10 %, 20 %, and 30 %). The addition of MSNs acted as a reinforcing agent, significantly improving the mechanical strength of the scaffolds-critical attribute for load-bearing applications in bone tissue engineering. This was achieved with only a negligible impact on the scaffold's high porosity, which is essential for nutrient transport and cell infiltration. The MSN-containing scaffolds exhibited a more controlled degradation profile, with degradation rates decreasing in an MSN concentration-dependent manner, suggesting greater stability in a physiological environment. From a biological standpoint, all fabricated scaffolds demonstrated excellent cytocompatibility. More importantly, the MSNs conferred a significant bioactive advantage. The scaffold containing 30 % MSNs (Alg/Chit/MSN30) not only was non-cytotoxic but also significantly promoted cell viability and proliferation compared to the Alg/Chit control. Critically for bone regeneration, the MSN-loaded composites exhibited vastly superior biomineralization capabilities *in vitro*, indicating a strong potential to support the formation of new bone mineral (Fig. 22C). In conclusion, this work effectively demonstrates the dual role MSNs can play within a single system: they function simultaneously as a structural nano-filler to improve mechanical integrity and as a bioactive component to stimulate a favorable cellular response and promote osteogenesis. The resulting Alg/Chit/MSN30 composite was identified as a highly promising candidate for bone tissue regeneration, serving as a prime example of how the strategic incorporation of MSNs can elevate a standard biopolymer scaffold into an advanced therapeutic material.

Moving beyond their role in reinforcing solid scaffolds, MSNs also offer sophisticated solutions for soft tissue engineering, particularly in hydrogel-based systems. For example, Rosenholm et al. formulated MSNs-based nanoparticle–hydrogel composites combining the benefits of bioactive signaling and cell-supportive delivery to optimize intracellular drug delivery for tissue regeneration [271]. The surface chemical properties of MSNs were precisely modulated to optimize their uniform distribution within three-dimensional matrices and to enhance the efficiency of intracellular drug delivery. Experimental results revealed that nanoparticle–hydrogel composites formulated with acetylated or succinylated MSNs exhibited outstanding spatial uniformity throughout the 3D matrix. Importantly, surface chemical modifications had a marked impact on cellular uptake kinetics: succinylated MSNs demonstrated rapid internalization, while acetylated MSNs displayed a sustained uptake profile. Moreover, the MSN–hydrogel nanocomposite system enabled highly efficient, localized intracellular delivery of hydrophobic model drugs (Fig. 22D and E). This work provides a compelling proof-of-concept for MSN-based surface engineering strategies, underscoring the considerable potential of this tunable drug delivery platform in tissue regeneration applications guided by bioactive signals, especially for the precise delivery of hydrophobic therapeutics.

## 6 Conclusions and perspectives

This review comprehensively summarizes the recent progress in nanotheranostic systems. It focuses on discussing their fundamental synthesis methods, functionalization strategies, and theranostic applications, aiming to offer insightful perspectives for the development of next-generation advanced nanotheranostic platforms.

As showcased throughout this review, MSN-based systems have demonstrated extraordinary performance outcomes in a wide array of preclinical models. For drug delivery, targeted modifications have enabled significant tumor accumulation, with some platforms achieving enrichment levels several-fold higher (*e.g.*, 4.7 times) than their non-targeted counterparts, thereby substantially improving local drug concentrations. In therapeutic applications, these platforms have achieved impressive results, such as attaining cure rates of up to 75 % in melanoma models through synergistic photothermal-immunotherapy and inhibiting tumor growth by over 80 % in other cancer models, while also effectively preventing recurrence and metastasis. For imaging, MSNs have served as versatile nanoprobes, enabling high-resolution *in vivo* imaging of inflammatory lesions and real-time monitoring of ion dynamics in the brain. These compelling preclinical results underscore the immense potential of MSNs and provide a strong foundation for their continued development toward clinical applications.

Initially, this review presents a fundamental discussion on the basic synthetic chemistry of MSNs, including the Sol-Gel method, Hydrothermal method, and Template method. Subsequently, the functionalized modification strategies of MSNs-based nanosystems are comprehensively elucidated. Crucially, our discussion has consistently framed these modifications not as isolated chemical techniques, but as essential tools for building theranostic function. These strategies include: (i) targeted modification, (ii) biomimetic modification, (iii) stimulus-responsive modification, and (iv) aptamer-based surface engineering. The corresponding functionalization mechanisms and guidelines are also extensively discussed. Finally, this review explores the applications of MSNs-based nanosystems in diagnosis and treatment. It anticipates collaborative efforts between researchers and engineers to expedite the clinical translation of these emerging platforms. We have showcased how these foundational and functionalization efforts converge to create powerful platforms for integrated diagnosis and treatment.

**Fig. 23:**
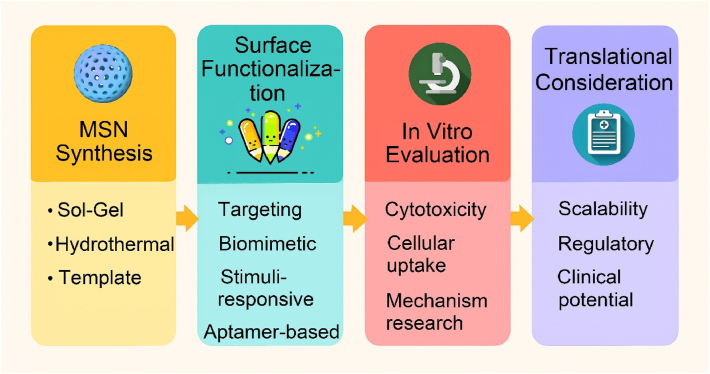
Schematic overview of the translational pipeline for MSNs. The diagram illustrates the sequential stages involved in the development of MSN-based nanoplatforms, including synthesis (*via* sol-gel, hydrothermal, or template methods), surface functionalization strategies (such as targeting, biomimetic, stimuli-responsive, and aptamer-based modifications), *in vitro* evaluation (covering cytotoxicity, cellular uptake, and mechanistic studies), and key translational considerations (including scalability, regulatory compliance, and clinical potential). This visual summary provides a concise and intuitive framework for understanding the progression from material design to biomedical application.

The entire developmental pathway, from initial synthesis to translational consideration, forms a comprehensive pipeline, as schematically illustrated in Fig. 23. This pipeline highlights the critical stages and decision points in the journey of an MSN-based system from a laboratory concept to a potential clinical product. However, advancing along this pipeline, particularly into the clinical realm, presents a unique set of challenges.

### 6.1 Clinical translation: hurdles and pathways forward

Despite the substantial progress in preclinical models, the translation of MSN-based platforms from the laboratory bench to clinical practice is fraught with significant hurdles. A realistic assessment of these barriers-spanning long-term toxicity, biodegradability, regulatory ambiguity, and a lack of robust human pharmacokinetic data-is crucial for guiding future research toward tangible medical applications. To contextualize these challenges, it is instructive to compare MSNs with established nanocarriers, such as liposomes and polymeric NPs (Table 5).

**Table 5:** Comparative analysis of drug-loading efficiency, stability, and clinical feasibility among nanocarriers.

Property	MSNs	Liposomes	Polymeric NPs
Drug-loading Efficiency	High (due to large surface area and tunable pore size)	Moderate (limited by lipid bilayer capacity)	Moderate to high (depends on polymer type and structure)
Stability	Excellent chemical and physical stability	Prone to leakage and fusion; limited physical stability	Good, but affected by polymer degradation and batch variability
Clinical Feasibility	Emerging; limited clinical trials; concerns about long-term safety and biocompatibility	Well-established; approved products and clinical use; biocompatible and biodegradable	Several approved products; scalable; biocompatibility varies by polymer type

Firstly, comprehensive evaluation of long-term biosafety remains a primary concern. While silica is generally regarded as safe (GRAS) by the FDA for oral consumption, the intravenous administration of nanosized silica particles presents a different toxicological profile. Key unresolved questions include: (i) Biodegradation and Clearance: The *in vivo* degradation rate of MSNs and the metabolic fate of their degradation products (orthosilicic acid) must be thoroughly understood, particularly the potential for nephrotoxicity if clearance pathways are overwhelmed [272]. (ii) Long-term Accumulation and Toxicity: Non-degraded or slowly-degrading MSNs can accumulate in organs of the reticuloendothelial system (RES), such as the liver and spleen. The potential for chronic inflammation, fibrosis, or other long-term pathological effects resulting from this accumulation is a critical safety issue that requires extensive investigation [273]. (iii) Immunogenicity: Despite surface modifications like PEGylation to create a “stealth" effect, MSNs can still be recognized by the immune system, potentially leading to complement activation and adverse infusion reactions. A systematic evaluation of the immunogenic potential of different MSN formulations is essential before clinical use [274].

Secondly, regulatory and manufacturing challenges pose a formidable barrier. The transition from lab-scale synthesis to large-scale, Good Manufacturing Practice (GMP)-compliant production is a major bottleneck for nearly all nanomedicines. For MSNs, ensuring batch-to-batch consistency in particle size, porosity, surface chemistry, and drug loading is technically demanding [275]. Furthermore, the regulatory pathway for complex nanotheranostic agents is not yet fully established. Regulatory bodies like the FDA and EMA require robust and standardized characterization protocols to define the critical quality attributes of the nanomaterial, a task complicated by the inherent complexity of multifunctional MSNs.

Finally, the landscape of clinical trials involving silica nanoparticles is still nascent. While a handful of silica-based nanoparticles have entered early-phase clinical trials, they are primarily simple formulations for imaging or drug delivery, not the complex, multi-functional theranostic systems envisioned in much of the research literature [32]. For instance, Cornell dots (fluorescent silica nanoparticles) have been tested in humans for cancer imaging, demonstrating a favorable safety profile. However, data from larger, later-phase trials demonstrating therapeutic efficacy for MSN-based systems are still largely absent. Overcoming the “valley of death" between promising preclinical data and successful clinical validation will require significant investment, cross-disciplinary collaboration between material scientists and clinicians, and intelligently designed clinical trials that target well-defined patient populations and clinical needs.

### 6.2 Future perspectives and challenges

Despite the substantial progress made, the continuous development of MSN-based biomaterials hinges on addressing several key challenges and embracing cutting-edge research directions. The future of this field will likely be defined by the convergence of materials science, biology, and data science, moving toward platforms that are more intelligent, personalized, and predictable.

1) Advanced Biohybrid MSN Designs

A pivotal frontier is the development of advanced hybrid MSN designs, moving beyond monolithic silica particles. While silica coating remains a versatile method for protecting nanomaterials like liquid metals or DNA origami [[276], [277], [278], [279], [280]], future research will focus on more deeply integrated systems. This involves creating biohybrid MSNs where the inorganic core is synergistically combined with biological components. Examples include MSNs with magnetic cores for MRI-guided therapy, MSNs templated by DNA origami scaffolds for unprecedented structural control [281], or coating with engineered cell membranes decorated with specific peptides (*e.g.*, “self" signals like CD47) for active immune modulation [[282], [283], [284], [285], [286], [287]]. Such platforms will offer a more nuanced biological identity, moving beyond passive “stealth" to achieve active biological functions.

2) “Smart" MSNs for Image-Guided and On-Demand Therapy

The next generation of MSNs will be increasingly “smart," capable of responding to specific biological cues with high precision. This involves refining stimuli-responsive release mechanisms to react to subtle changes in the tumor microenvironment (*e.g.*, specific enzyme levels, redox state). Furthermore, integrating diagnostic and therapeutic functions will enable true image-guided delivery, where real-time feedback on nanoparticle accumulation and therapeutic response can inform treatment decisions. The rise of “nano-catalytic medicine," where MSNs act as nanoreactors to promote therapies like PDT, SDT, and CDT, is a prime example of this trend, transforming MSNs from passive carriers into active therapeutic participants.

3) Expanding Therapeutic Horizons: Beyond Oncology to Regenerative and Neuroprotective Medicine

The versatility of MSNs is underscored by their expanding applications in areas like “nano-catalytic medicine" and regenerative therapies. While broadly utilized in oncology (*e.g.*, PDT, SDT, CDT), the utility of MSNs now extends to several other critical fields. For instance, in neurodegenerative diseases, they are being engineered to traverse the blood-brain barrier, delivering neuroprotective agents or scavenging harmful ROS implicated in neuronal damage. Furthermore, in regenerative medicine, MSNs exhibit significant promise for accelerating wound healing by enabling the sustained, localized delivery of growth factors, antimicrobial agents, or even oxygen to the wound bed. These emerging applications, alongside their established roles in anti-inflammatory, antibacterial, and vaccine development, highlight the platform's remarkable adaptability. Considering their intrinsically adjustable mesoporous structures, MSNs facilitate the exchange of substances between the internal and external environments, promoting these diverse therapeutic actions. Therefore, the continued design of multifunctional MSN-based platforms is expected to expedite progress across this broad therapeutic landscape.

4) MSNs as a Platform for Synergistic Immuno-Oncology Therapies

Recent oncology researches have placed emphasis on integrating conventional therapies (such as chemotherapy, PDT, CDT) with immunotherapy to synergistically boost therapeutic efficacy. This has propelled the progress of chemoimmunotherapy, photoimmunotherapy, and radioimmunotherapy [[288], [289], [290], [291], [292], [293]]. This trend has notably advanced the design of immunomodulatory nanosystems, creating favorable conditions for the integration of various therapeutic modalities [[294], [295], [296]]. For instance, recent studies have demonstrated that mesoporous silica and organosilica nanoparticles can serve as powerful platforms for cancer immunotherapy by co-delivering antigens and adjuvants to trigger potent systemic anti-tumor immunity [297]. Similarly, engineering hollow organosilica networks to respond to the tumor microenvironment enables sustained dynamic therapies, showcasing a sophisticated strategy to overcome treatment resistance [298]. However, the immunogenic potencies of MSNs have not been systematically evaluated to date, and research on MSN-based immunotherapy is still in its infancy. Thus, we expect the development of more viable MSN-based nanosystems to facilitate future immunotherapy.

5) Toward Personalized Nanomedicine

The ultimate goal of nanomedicine is personalization. The versatility of MSN surface chemistry provides an ideal platform for developing personalized nanomedicine. Future MSNs could be tailored to a patient-specific tumor signature. For instance, aptamers or antibodies targeting unique biomarkers identified from a patient's biopsy could be grafted onto MSNs. In combination with technologies like CRISPR, MSNs could be designed to deliver gene-editing tools that correct patient-specific mutations, opening the door to truly individualized therapeutic strategies.

6) AI-Guided Design and Optimization

Finally, the traditional trial-and-error approach to nanomaterial design is slow and inefficient. The emergence of Artificial Intelligence (AI) and machine learning offers a transformative path forward. By building large datasets that correlate MSN physicochemical properties (size, charge, surface chemistry) with biological outcomes (pharmacokinetics, toxicity, efficacy), AI-guided design can predict the performance of novel platforms in silico. This will dramatically accelerate the optimization of MSN formulations, helping researchers navigate the vast design space to identify candidates with the highest potential for successful clinical translation.

7) Bridging the Gap: Challenges and Strategies for the Clinical Translation of MSNs

Clinical translation is the ultimate goal in the advancement of cutting-edge nanomedicine [299]. Regrettably, current research on the design and fabrication of multifaceted MSNs is still mainly limited to preliminary experimental phases in existing small animal models. Although these studies offer substantial preclinical evidence (such as hematological and histological parameters) to demonstrate efficacy and safety, their translational significance for human physiology remains unclear [300]. A primary hurdle in this regard is the long-term biosafety profile of MSNs. The inherent non-biodegradability of the conventional silica framework raises significant concerns about potential chronic toxicity due to its gradual accumulation in organs of the reticuloendothelial system, such as the liver and spleen. Therefore, addressing these safety issues is paramount for clinical translation. Current strategies to mitigate these risks are multifaceted. One approach involves precisely tailoring the particle size to be below the renal clearance threshold (<10 nm) to facilitate efficient excretion. Another promising direction is the development of biodegradable MSNs, where cleavable bonds are incorporated into the silica matrix, allowing the nanoparticles to decompose into harmless, excretable silicic acid byproducts within the physiological environment. Furthermore, advanced surface engineering strategies remain critical to modulate *in vivo* behavior and minimize off-target accumulation. Closing the gap between laboratory research and clinical application remains a crucial priority for all researchers. Thus, close collaboration among universities, research institutions, and hospitals is essential to promote the clinical translation of MSNs and drive the progress of future medicine.

From a materials perspective, the intrinsic structural features, physicochemical properties, and nano-bio interactions of MSNs render them a versatile nanoplatform for material-based therapeutic applications. They have spurred collaborative efforts among pharmacologists, materials scientists, and clinicians. These professionals are dedicated to designing, fabricating, and optimizing these novel nanosystems, ultimately providing superior therapeutic options for clinical use. The past few decades have witnessed the prominent advancements of MSNs in the biomedical field. It is anticipated that in the next decade, true transformative breakthroughs will emerge not from incremental advances, but from the rational convergence of key strategies designed to overcome the aforementioned translational hurdles. This includes creating intelligent hybrid designs (*e.g.*, hybrid organic-inorganic shells) and biodegradable frameworks (*e.g.*, incorporating cleavable bonds) to resolve long-term toxicity, and developing precisely engineered surfaces for immune modulation to improve safety and efficacy. Ultimately, these material science innovations, coupled with intelligently designed clinical trials, will be essential to finally bridge the gap to clinical reality.

## CRediT authorship contribution statement

**Yuying Liu:** Writing – original draft, Resources, Formal analysis, Data curation. **Man Zhao:** Writing – original draft, Validation, Methodology. **Meihua Zhang:** Writing – review & editing, Supervision, Conceptualization. **Bin Yang:** Resources. **Yun-Kun Qi:** Writing – review & editing, Funding acquisition. **Qinrui Fu:** Writing – review & editing, Supervision, Software, Resources, Project administration, Methodology, Funding acquisition, Conceptualization.

## Declaration of competing interest

There are no conflicts to declare.

## Data Availability

No data was used for the research described in the article.
